# Diet Quality and Nutrient Adequacy Among Polish Children: Findings from the PITNUTS 2024 Study

**DOI:** 10.3390/nu17213364

**Published:** 2025-10-26

**Authors:** Michał Sawicki, Joanna Kowalkowska, Ewa Kawiak-Jawor, Zbigniew Kulaga, Grazyna Rowicka, Piotr Socha, Anna Swiader-Lesniak, Agnieszka Swiecicka-Ambroziak, Hanna Szajewska, Lidia Wadolowska, Malgorzata Wiech, Halina Weker

**Affiliations:** 1 Nutricia Foundation, 8 Bobrowiecka Street, 00-728 Warsaw, Poland; michal.sawicki@fudacjanutricia.pl; 2 Department of Human Nutrition, Faculty of Food Science, University of Warmia and Mazury in Olsztyn, 45F Sloneczna Street, 10-718 Olsztyn, Poland; lidia.wadolowska@uwm.edu.pl; 3 Department of Social Innovation, Łukasiewicz Research Network—ITECH Institute of Innovation and Technology, 87 Zelazna Street, 00-879 Warsaw, Poland; ewa.kawiak-jawor@itech.lukasiewicz.gov.pl; 4 Department of Public Health, Children’s Memorial Health Institute, 20 Dzieci Polskich Avenue, 04-730 Warsaw, Poland; z.kulaga@ipczd.pl; 5 Pediatric Gastroenterology Outpatients Clinic, Institute of Mother and Child, 17a Kasprzaka Street, 01-211 Warsaw, Poland; grazyna.rowicka@imid.med.pl; 6 Department of Gastroenterology, Hepatology, Nutritional Disorders and Paediatrics, Children’s Memorial Health Institute, 20 Dzieci Polskich Avenue, 04-730 Warsaw, Poland; p.socha@ipczd.pl; 7 Department of Anthropology, Children’s Memorial Health Institute, 20 Dzieci Polskich Avenue, 04-730 Warsaw, Poland; a.swiader-lesniak@ipczd.pl; 8 Medical Affairs and Market Access Department, Nutricia Polska, 8 Bobrowiecka Street, 00-728 Warsaw, Poland; agnieszka.swiecicka@danone.com; 9 Department of Paediatrics, Medical University of Warsaw, 61 Żwirki i Wigury Street, 02-091 Warsaw, Poland; hanna.szajewska@wum.edu.pl; 10 Department of Nutrition, Institute of Mother and Child, 17a Kasprzaka Street, 01-211 Warsaw, Poland; malgorzata.wiech@imid.med.pl (M.W.); halina.weker@imid.med.pl (H.W.)

**Keywords:** children, dietary inadequacies, dietary patterns, nutrition, Poland

## Abstract

Background/Objectives: The past evidence indicates that Polish children’s diets frequently deviate from recommendations. The aim of the PITNUTS 2024 study was to provide an updated nationwide assessment of energy and nutrient intake among children aged 5–72 months, evaluate the risk of inadequate intake, and examine the relationship between diet quality patterns and nutritional adequacy. Methods: PITNUTS 2024 was a cross-sectional study analyzing dietary data from a representative sample of 940 Polish children. Dietary intake was assessed qualitatively and quantitatively. Nutrient adequacy was evaluated using the estimated average requirement or adequate intake cut-point method. Two diet quality scores were developed: the Children’s pro-Healthy Diet Score and the Children’s non-Healthy Diet Score, and their association with the risk of inadequate intake was evaluated using logistic regression. Results: The proportion of energy derived from protein intake exceeded recommended levels in most children, while that from fat was typically below reference levels, especially in younger groups of children. The risk of inadequate energy intake from carbohydrates was uncommon, while sucrose intake exceeding 10% of overall energy was present in almost half of the children. Among children aged 13–72 months, approximately 15% adhered to high child-pHDS, associated with a lower risk of insufficient intake of selected nutrients. Conclusions: The diets of Polish children aged 5–72 months show persistent nutritional risks, including excessive protein intake, low vitamin D intake, suboptimal fatty acid intake profiles, and insufficient calcium and fibre intake. Diet quality scores can be useful for identifying children at risk of inadequate nutrient intake.

## 1. Introduction

Optimal nutrition in early childhood is crucial for healthy growth, psychomotor development, and the prevention of diet-related diseases. The concept of the “first 1000 days” emphasizes that appropriate feeding practices from fetal life through the first two years establish the foundation for long-term health. Key infant feeding recommendations include exclusive breastfeeding for the first six months of life, timely introduction of complementary foods, and continued breastfeeding alongside diversified diets into toddlerhood [1,2,3].

However, evidence from Poland has shown substantial deviations from recommended feeding practices. Studies conducted in 2011 and 2016 revealed significant imbalances in young children’s diets [4,5]. The Polish Infant and Toddler Nutrition study (PITNUTS) on a representative sample of 1059 infants and toddlers (5–36 months old) found low rates of exclusive breastfeeding up to six months, and a high proportion of abnormal weight status among infants and toddlers [5,6]. These findings underscore the need for improved nutritional guidance and interventions for families.

Similar dietary inadequacies in early childhood have been reported globally, underlining the importance of this issue for the broader public health [7,8,9,10,11]. In recent years, international and national authorities have updated their dietary recommendations for infants and young children. Notably, the European Society for Pediatric Gastroenterology, Hepatology and Nutrition (ESPGHAN) issued new guidance on complementary feeding and related practices [12], including the introduction of allergenic foods, caution against the use of plant-based milk alternatives in infancy, and the avoidance of fruit juices in the first year of life. In Poland, infant nutrition guidelines revised in 2021 align with these standards [13]. Updated model food ration recommendations for children aged 1–9 years have also been introduced to promote balanced diets in line with current evidence [13].

Unhealthy diets in childhood contribute to the growing burden of obesity and related non-communicable diseases [10,14]. Poor diet quality in early childhood is associated not only with immediate issues like nutrient deficiencies but also with higher risks of overweight in later life [15,16,17]. Monitoring and improving young children’s diet quality has become a public health priority [10]. Diet quality patterns became an important dimension to dietary assessment [18]. A composite score based on the frequency of consumption of healthy or unhealthy food groups provides a structured way to evaluate diet quality and link to nutrition status or health outcomes.

PITNUTS 2024 was designed as a nationwide cross-sectional survey to provide an updated assessment of dietary practices and nutritional status among Polish children aged from 5 months to 6 years. Its primary objective is to characterize the intake of macronutrients (protein, fat, and carbohydrates) in infants and young children. Secondary objectives include evaluating the nutritional value of the diet in terms of other nutrients and estimating the proportion of children at risk of insufficient intake in relation to dietary standards. The PITNUTS 2024 study also assessed the prevalence of underweight, overweight, or obesity across age groups, based on anthropometric measurements. The present study aimed to evaluate the prevalence of inadequate nutrient intake compared to dietary recommendations among Polish children aged 5–72 months as well as to examine the diet quality using validated dietary scores and their association with nutritional adequacy. By evaluating adherence to updated recommendations, the PITNUTS 2024 study aims to identify persistent gaps in early childhood nutrition and generate evidence to guide targeted improvements in dietary policy and parental education.

## 2. Materials and Methods

PITNUTS 2024 was a nationwide, cross-sectional survey designed to assess dietary practices and nutritional status in a representative sample of Polish children aged 5 to 72 months. The study was conducted between September—December 2024 using stratified random sampling to ensure representation across child age groups and regions of Poland.

The study was approved by the Ethics Committee of the Institute of Mother and Child in Warsaw. Written informed consent was obtained from all participating parents or legal guardians. Sampling and recruitment were performed by Minds & Roses Ltd., Warsaw, Poland. The study is registered with ClinicalTrials.gov, NCT06417151: https://clinicaltrials.gov/study/NCT06417151 (accessed on 16 May 2024).

### 2.1. Sampling

A stratified, multistage random sampling method was used to ensure representativeness by region, municipality type, and child age group. Primary sampling units were municipalities, selected with probability proportional to the number of children aged under 6 years using data from the 2021 National Population Census [19]. To draw the primary sampling units from the set of Polish municipalities, four strata were constructed based on the type of municipality: rural, urban–rural, urban ≤100,000 inhabitants, and urban >100,000 inhabitants.

Within each selected municipality, children were randomly selected, using a probability proportional to the number of children under six residing in each municipality, based on their national identification numbers (PESEL). In cases where a selected municipality had an insufficient number of children in the target age group, the nearest municipality within the same stratum was added to the cluster to ensure an adequate sample size. The final sample included 1000 children, stratified as follows:

- 300 infants aged 5–12 months (subgroups: 5–6, 7–9, and 10–12 months; *n* = 100 each);
- 400 children aged 13–36 months (subgroups: 13–24 and 25–36 months; *n* = 200 each);
- 300 children aged 37–72 months (subgroups: 37–48, 49–60, and 61–72 months; *n* = 100 each).

The territorial representation of the drawn sample in voivodships and municipalities of various types is presented in Table S1. To address potential declines in participation and respondent unavailability on the survey date, two parallel cluster samples of municipalities were drawn, each with allocated age groups to achieve a defined distribution of responses across ages.

### 2.2. Inclusion and Exclusion Criteria

The primary respondents were the parents responsible for the child’s care and nutrition. Eligible participants were healthy, term-born children of Polish nationality aged 5–72 months. Children were excluded if they had chronic conditions affecting nutritional status (e.g., metabolic, gastrointestinal, kidney, liver diseases), developmental disorders (e.g., genetic syndromes, prematurity), acute infections or surgeries within the preceding 3 months, prolonged hospitalization (>10 days) or surgeries which could affect nutritional status, or use of medically indicated elimination diets (e.g., allergies).

### 2.3. Endpoints

The primary endpoint of the study was to assess the nutritional value of the diet in terms of macronutrient intake, specifically protein, fat, and carbohydrates.

Nutritional value of the diet in terms of additional dietary components, including animal and plant proteins, carbohydrates, fibre, lactose, sucrose, starch, polyunsaturated fatty acids (PUFA), monounsaturated fatty acids (MUFA), and saturated fatty acids (SFA), vitamins, and minerals, was the secondary endpoint. Other secondary endpoints included evaluation of population-level risk of inadequate nutrient intake using the estimated average requirement (EAR) or adequate intake (AI) cut-point method, with particular focus on vitamin D, long-chain polyunsaturated fatty acids (LCPUFA), and protein, as well as the assessment of the nutritional status of children using body mass index (BMI) z-score based on anthropometric measures, including body weight, and length/height. In this report, the former endpoint is presented only in the context of study group characteristics solely to demonstrate that the described dietary patterns pertain to a sample of children with a defined nutritional status. The endpoint will be the topic of a separate publication.

Among the exploratory endpoints, this report includes analyses of associations between predefined dietary patterns (diet quality scores) and the nutritional value of the diet. Certain socio-demographic factors (sex and age of children, education level of parents), the child’s BMI z-score, and the BMIs of parents were used as covariates in assessing the above associations.

### 2.4. Study Sample

The average intake of macronutrients, the primary endpoint, informed sample size estimation. The subgroup of infants aged 5–6 months was selected for reference due to the highest observed variability in macronutrient intake in the previous PITNUTS 2016 study [5]. Based on those data, the minimum required sample sizes to estimate mean intake with a 15% allowable margin of error were calculated as follows: 97 for protein, 91 for fat, and 94 for carbohydrates. Accordingly, a conservative target of 100 infants in this age subgroup was set. Similar subgroup sizes were adopted a priori across other age groups to allow estimation of nutrient intakes with an acceptable margin of error of 5–10%. Beyond estimating mean intakes, the sample supported the assessment of nutrient inadequacies and stratified anthropometric status. The sampling plan maintained a 95% confidence interval and a maximum margin of error of 6% for prevalence estimates within each major age category.

The sample design incorporated proportional allocation across four strata of municipality types, based on the number of children aged under 6 years [19]. This approach ensured territorial representativeness not only for the national sample as a whole but also within each age cohort. The use of cluster sampling (sampling by municipality) optimized logistical efficiency while preserving the statistical validity of estimates through appropriate weighting and variance estimation. The territorial structure of the implemented parallel samples was the same for each cohort (Table S1). This ensured territorial representativeness of the sample not only for the entire study but also for each age cohort individually.

### 2.5. Data Collection and Measures

Interviewers, trained by dieticians and anthropologists, conducted structured interviews with parents or legal guardians of infants and children. Retail shop vouchers were proposed as an incentive to encourage participation. The assessment included a collection of socio-demographic, dietary, and anthropometric data.

#### 2.5.1. Socio-Demographic, Lifestyle and Anthropometric Data

Socio-demographic and lifestyle information about infants, children, and their parents were collected via standardized questionnaires using a computer-assisted personal interview (CAPI) system. Variables used to characterize the study sample included: sex and age of children, type of municipality, economic situation of household, parental education levels, nutrition knowledge of parent-proxy reporter, and child’s physical activity level compared to peers.

Anthropometric measurements, including body weight and length/height, were conducted using Seca instruments (Seca, Germany, Hamburg): Seca 834 and 417 for infants (weight and length), and Seca 878 and 217 for older children (weight and height). Measurements followed the World Health Organization (WHO) guideline [20]. BMI-for-age z-scores using measured length/height and weight were calculated, and categorized according to the WHO standards suitable for children aged 5–60 and 61–72 months into four categories of nutritional status, i.e., underweight, normal weight, overweight, and obesity [21,22]. Analysis was performed using WHO Anthro 3.2.2.1 and WHO AnthroPlus 1.0.4 software (WHO, Geneva).

#### 2.5.2. Assessment of Diet Quality

Qualitative assessment employed three age-specific dietary questionnaires adapted from the PITNUTS 2016 study [5] and administered via a CAPI system. These questionnaires included the food frequency questionnaire (FFQ) and were validated for the test–retest reproducibility with 100 participants per age group.

Qualitative data on the frequency of consumption of different food groups during the last month were collected via the 85-item Child Food Frequency Questionnaire (85–item Child–FFQ). This qualitative FFQ included a cafeteria of six frequency categories to choose from, which have been converted into daily consumption frequency using the coefficients given in brackets: never or almost never (0 times/day), less than once a week (0.06), once a week (0.14), 2–6 times a week (0.57), once a day (1), several times a day (2).

To facilitate a comprehensive assessment of diet quality among children aged 13 to 72 months, two predefined dietary patterns were developed based on an extensive review of the literature and existing dietary guidelines: (i) the Children’s pro-Healthy Diet Score (child–pHDS) and (ii) the Children’s non-Healthy Diet Score (child–nHDS).

The child–pHDS was developed to capture the frequency of consumption of food groups associated with pro-healthy dietary habits and comprises seven food groups represented by 25 food items from the 85-item Child–FFQ: vegetables, pulses/nuts, fruits, cereal products, unsweetened dairy products, unprocessed animal-source foods (meat, fish, eggs), and recommended fats (e.g., vegetable oils).

In contrast, the child–nHDS was developed to capture the frequency of consumption of food groups associated with unhealthy dietary habits and comprises ten food groups encompassing 28 food items: sweetened breakfast cereals, fried flour-based dishes (e.g., pancakes, dumplings), fast food, sweetened dairy products, processed meat, food concentrates/salty seasonings, sweet condiments (e.g., sugar, jam), sweets, salty snacks, and sweetened beverages. For each food group, daily consumption frequency (times/day) was computed by summing the reported frequencies of its component food items. Median daily consumption frequencies were calculated separately for two age groups: 13–36 months and 37–72 months. If a child’s consumption of a given food group exceeded the age-specific median, one point was assigned. The total score of each diet quality score was the sum of food groups with above-median consumption.

Therefore, a total child–pHDS ranged from 0 to 7 points, and was categorized into three levels reflecting increasing intensity of pro-healthy dietary behaviours: low (0–2 points), moderate (3–5 points), and high (6–7 points). A total child-nHDS ranged from 0 to 10 points, and was categorized into three levels, indicating increasing intensity of unhealthy dietary behaviours: low (0–3 points), moderate (4–7 points), and high (8–10 points). Detailed information on the components of the diet quality scores and their scoring is given in the Tables S2 and S3.

#### 2.5.3. Assessment of Nutritional Value of the Diet

Quantitative dietary data, based on 3-day food record covering three consecutive days (including one weekend day), were completed by parent-proxy reporter for their children. Assessment was performed using either paper-and-pencil interviewing or online methods. Parent-proxy reporters received detailed instructions with examples on how to complete the 3-day food record, including the examples of portions of various foods and meals. The amount of food was assessed using household measures or in grams. If there were any questions related to filling out the food record, parent-proxy reporters could also contact a nutritional consultant. Nutrient intakes were analyzed using the DIETA 6.0 software (National Institute of Public Health, Poland), referencing national food composition tables. Population-level risk of inadequate nutrient intake was evaluated using the cut-point method based on the EAR or AI. Additional thresholds, such as sucrose intake exceeding 10% of daily energy intake [23], were applied to identify dietary risks. In total, energy and intake of 29 nutrients were analyzed. The recommended nutrient intake was based on the Nutrition Standards for the Polish Population [24] and WHO recommendations [23,25,26]. The dietary standards categorized by age groups are presented in Table S4.

### 2.6. Statistical Analysis

Data collected during the study were analyzed using descriptive statistics, including counts and percentages for variables, and medians with lower and upper quartile ranges (Q1–Q3) and quartile coefficient of variation (CV_Q_ = (Q3 − Q1)/median × 100%) for continuous variables. The following statistical tests were used to assess the significance level: the Mann–Whitney test or the Kruskal–Wallis test for continuous variables and the Chi^2^ test for categorical variables. To account for multiple testing, the Benjamini–Hochberg false discovery rate (FDR) correction was applied to all *p*-values.

Logistic regression analysis was applied to assess the association between the diet quality scores, child-pHDS and child-nHDS (high vs. low levels), and the risk of inadequate intake of analyzed nutrients (adequate vs. inadequate intake). The reference groups were children with a high level of each diet quality score or adequate nutrient intake. Crude and adjusted odds ratios (ORs) with 95% confidence intervals (95% CIs) were calculated. The ORs were adjusted for seven variables: sex, age, and BMI-for-age z-score of children, as well as education levels and BMIs of mother and father. The Wald’s statistics was used to assess the significance level of ORs.

Apparent gaps in variables, such as paternal education, were coded in the questionnaire as a specific category (“no information”) and analyzed accordingly. No data were missing in the FFQ section used to calculate diet quality scores, and all 3-day food records were completed for children consuming any food or beverages. As part of data quality control, 60 children with a mean daily energy intake <14 kcal/day and 31 children with implausible anthropometric values (identified using WHO Anthro/AnthroPlus software) were excluded from relevant analyses. All analyses were conducted on complete cases.

Data were analyzed using Statistica 13 (TIBCO Software Inc., Tulsa, OK, USA; StatSoft Polska, Cracow, Poland) and PS IMAGO PRO 10.0 on the IBM SPSS Statistics 30 analytical engine (Predictive Solutions, Cracow, Poland). *p*–values below 0.05 were considered statistically significant.

## 3. Results

**Figure 1:**
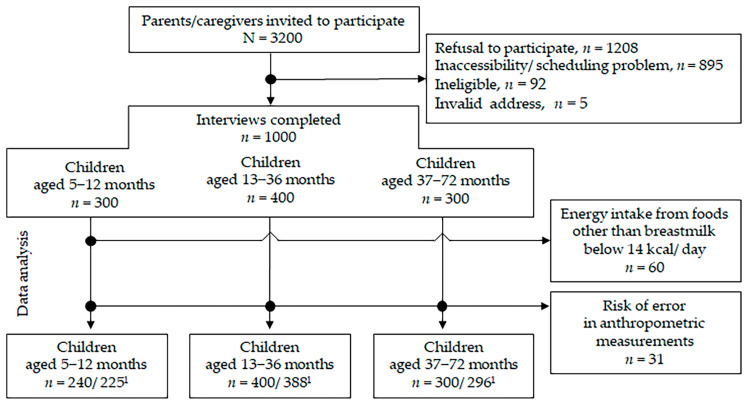
Flow diagram for study participants. ^1^ indicates total sample size analyzed/ sample size with reliable anthropometric measurements.

To conduct 1000 planned interviews, 3025 parents or legal guardians were contacted. The main reasons for non–response were refusal to participate (54.9%) or inaccessibility or scheduling problem (40.7%) (Figure 1). At the time of the study, 190 of 300 infants aged 5–12 months (63.3%) were being breastfed. In this age group, there were 60 breastfed infants with energy intake from foods other than breast milk of less than 14 kcal/day. These infants were excluded from further analysis (Figure 1). Consequently, the analyzed group of breastfed infants aged 5–12 months comprised 130 participants. Finally, the total study sample included 940 children (Table 1). Extreme deviations in the standardized indicator ranges were present in the case of 31 children, who were excluded from further analysis due to a high risk of errors in anthropometric measurements (Figure 1, Table 1).

### 3.1. Characteristics of the Study Sample

The socio–demographic and lifestyle characteristics of participants are presented in Table 1. In each age group, females constituted a slight majority. Considering the place of residence, almost one third of the children from each age group lived in rural areas, nearly a quarter of the children lived in urban–rural areas, and approximately 45% of children lived in urban areas. The parents of the children most often had secondary or higher education. Mothers more frequently hold higher education degrees compared to fathers. Most parents assessed their household’s financial situation as good. Over two–thirds of parents declared that their knowledge of nutrition principles is good.

### 3.2. Energy and Nutrient Intake

The daily energy content of complementary feeding (including formula) among breastfed infants varied significantly (median, 372.9; Q1–Q3, 164.4–631.7). The median intake of protein from complementary foods to breastfeeding was 11.6 g (Q1–Q3, 5.2–21.3), fat 11.6 g (Q1–Q3, 3.9–19.0), and carbohydrates 57.1 g (Q1–Q3, 27.8–97.8). The median proportion of energy derived from protein, fat, and carbohydrates was 13.1%, 25.7%, and 57.8%, respectively.

Table 2 presents the macro- and micronutrient intake in groups of children aged 5–12 months (non-breastfed only), 13–36 months, and 37–72 months. The daily median protein, fat, and carbohydrate content was 19.4 g (Q1–Q3, 14.7–25.4), 25.5 g (Q1–Q3, 20.5–30.8), and 109.1 g (Q1–Q3, 87.7–133,4), respectively. The median percentage of energy derived from macronutrients was 10.5% from protein, 31.6% from fat, and 54.8% from carbohydrates.

The daily median protein, fat, and carbohydrate content in the group of children aged 13–36 months was 38.5 g (Q1–Q3, 28.4–47.8), 34.1 g (Q1–Q3, 24.5–41.0), and 142.0 g (Q1–Q3, 114.2–174.6), respectively. Median 15.5% of energy derived from protein, 28.6% from fat, and 53.2% from carbohydrates. In the group of preschool children (37–72 months), the median protein, fat, and carbohydrate intake was 50.3 g (Q1–Q3, 41.7–59.3), 41.4 g (Q1–Q3, 34.1–50.5), and 172.0 g (Q1–Q3, 147.9–206.9), respectively. In this group, the median percentage of energy derived from macronutrients was 15.8% from protein, 29.6% from fat, and 52.7% from carbohydrates (Table 2).

### 3.3. Risk of Inadequate Nutrient Intake

The proportion of children in each age group at risk of inadequate nutrient intake is presented in Table 3. The differences in the proportion of children at risk of inadequate intake of the analyzed nutrients between the three age groups were statistically significant (*p* < 0.05), except for potassium and vitamins A, B_6_, and B_12_.

All non-breastfed children aged 5–12 months were at risk of inadequate intake of energy derived from fat. The risk of inadequate intake of energy from carbohydrates, i.e., below acceptable macronutrient distribution range (AMDR), was uncommon. Sucrose intake exceeding 10% of overall energy was present in 400 children (49.4%), and was most common in the oldest group (Table 3).

The risk of deficiency of vitamin D was common across all age groups and present in all children aged 37–72 months. The risk of LCPUFA deficiency was increasing with age (*p* < 0.001). Intake of EPA and DHA was inadequate in the majority of children participating in the study (Table 3).

### 3.4. Diet Quality and the Risk of Inadequate Nutrient Intake

Diet quality patterns among children in the post-infancy and preschool period were similar. The child–pHDS indicated a low intensity of pro-healthy dietary characteristics in more than one-third of the children, moderate intensity in almost half, and high intensity only in every sixth or seventh child (Table 4). 

The child–nHDS indicated a high intensity of unhealthy dietary characteristics that were observed in 14.2% of children in the younger age group (13–36 months) and in 13.7% of children in the older age group (37–72 months) (Table 4). The daily energy and nutritional value of the diet varied across the diet quality levels, especially in children aged 13–36 months (Tables S5 and S6). For example, among children aged 13–36 months with higher child-pHDS level, there was a significantly higher intake of most vitamins and minerals, except iodine, vitamin A, β-carotene, vitamin D, E and C compared to children with low child-pHDS (Table S5). However, these associations were not present in the older group (Table S6).

The diet quality levels in children aged 13–72 months were associated with the risk of inadequate nutrient intake according to the dietary recommendations. The percentage of children aged 13–36 and 37–72 months at risk of inadequate nutrient intake according to the dietary recommendations by the diet quality levels is presented in Tables S7 and S8. For example, among children aged 13–36 months with higher child-pHDS level, there was a significantly lower percentage of children at risk of inadequate intake of protein, fibre, copper or B vitamins compared to children with low child-pHDS (Table S7). However, no significant associations were found among children aged 37–72 months (Table S8).

Odds ratios (ORs) for the risk of inadequate nutrient intake, adjusted for confounding factors, in relation to the diet quality levels in children aged 13–72 months are presented in Table 5. Compared to children aged 13–36 months with low child–pHDS (the reference group), those with high level of child–pHDS were significantly more likely to have percentage of energy intake from protein above acceptable macronutrient distribution range (AMDR) (OR_adj_ = 2.73, *p* < 0.05) and percentage of energy intake from carbohydrates below AMDR (OR_adj_ = 3.14, *p* < 0.05), while significantly less likely to have insufficient intake of EPA and DHA (OR_adj_ = 0.22, *p* < 0.01), fibre (OR_adj_ = 0.39, *p* < 0.01), calcium (OR_adj_ = 0.45, *p* < 0.05), vitamin B_1_ (OR_adj_ = 0.20, *p* < 0.05), niacin (OR_adj_ = 0.19, *p* < 0.01), folates (OR_adj_ = 0.24, *p* < 0.01), and vitamin C (OR_adj_ = 0.24, *p* < 0.05). Compared to children aged 13–36 months with low child–nHDS (the reference group), those with the high level of child–nHDS were significantly less likely to have a percentage of energy intake from ALA below AI (OR_adj_ = 0.41, *p* < 0.05). Compared to children aged 37–72 months with low child-pHDS (the reference group), those with the high level of child–pHDS were significantly less likely to have a percentage of energy intake from sucrose above 10% (OR_adj_ = 0.41, *p* < 0.05) and insufficient intake of calcium (OR_adj_ = 0.16, *p* < 0.01). Compared to children aged 37–72 months with low child-nHDS (the reference group), those with high level of child-nHDS were significantly more likely to have: percentage of energy intake from ALA below AI (OR_adj_ = 2.55, *p* < 0.05), insufficient intake of fibre (OR_adj_ = 3.44, *p* < 0.05) and magnesium (OR_adj_ = 10.35, *p* < 0.05). Crude ORs for the risk of inadequate nutrient intake according to the dietary recommendations by the diet quality levels in children aged 13–72 months are presented in Table S9. It is worth noting that the ORs obtained, for example, for sodium turned out to be insignificant after taking into account the influence of other factors, such as sex, age and BMI-for-age z-score of children, as well as parental education levels and BMIs.

## 4. Discussion

This nationwide cross-sectional study provides an updated and comprehensive assessment of dietary intake and nutritional status among Polish children aged 5 to 72 months. The findings confirm the persistence of previously reported nutritional risks and identify age-specific patterns of dietary inadequacy and excess. The dietary practices of the children under study exhibited significant deviations from established principles of safe nutrition. Notable discrepancies were identified in breastfeeding practices, the timing and methods of introducing complementary foods, and the overall energy and nutritional composition of the diets. Compared with the results of the PITNUTS 2016 study [5], the data from 2024 demonstrate some areas of improvement, while highlighting continued challenges that require public health attention.

### 4.1. Macronutrient Intake

The analysis of macronutrient intake revealed that, although total energy intake generally met or exceeded age-specific requirements, there were substantial deviations from recommended macronutrient distribution across all age groups. Most notably, protein intake significantly exceeded dietary reference values in the majority of children, continuing the trend reported in PITNUTS 2016 [5]. In the current study, the risk of inadequate protein intake was rare, observed in approximately every tenth non-breastfed infant, and was almost nonexistent after infancy and in the preschool age. In these groups, the median protein intake was approximately three times higher than the EAR. Similarly, elevated protein intakes have been reported in other European cohorts [10,27]. No European national survey has found average protein consumption to be below the recommendations; therefore, protein deficiency is virtually nonexistent in this age group in developed countries [10]. In our study, only 3% of children had protein consumption below AI or EAR. A pattern of excessive protein intake in early childhood was already evident in infancy and remained stable into the preschool years, raising concerns regarding long-term metabolic programming and an increased risk of obesity [28,29]. Excessive protein intake during early childhood is a widespread issue across Europe, and the primary challenge lies in aligning intake with recommended levels.

Fat intake was highly variable and was below reference values in half of the analyzed children. Energy from the fat intake below the reference range was present in all non-breastfed infants, and the majority of children aged 13–36 months. The contribution of SFA often exceeded recommended limits (<10% of total energy), particularly among non-breastfed infants and preschoolers. The previous study among Polish preschool children revealed an excessive intake of SFA in 95% of children [30]. In the pan-European review, in all examined countries, children and adolescents exceeded the recommended <10% of energy from SFA [10]. Similarly, a global review reported that SFA intake equal to or exceeding 10% of total energy occurred in 69–73% of children. Notably, the review confirmed that in all European countries, across all age groups, SFA consumption exceeded the recommended threshold [31]. Conversely, PUFA, especially omega-3 fatty acids (EPA + DHA), were underrepresented in the diet across all subgroups [10,31]. These findings of suboptimal omega-3 intake are in line with broader European data that highlight insufficient consumption of fishery and aquaculture products, indicating a general decline in their consumption [32].

Observed patterns of carbohydrate intake and sugar consumption in Poland also reflect negative international trends. Total carbohydrate as a share of energy tends to be adequate in infancy and declines in older children. Interestingly, every tenth child aged 13–72 months had a lower-than-recommended percentage of energy from carbohydrates. An alarming finding was the high intake of sucrose, especially in the 13–36 and 37–72 months groups, where 50% or more of children exceeded the 10% total energy threshold. A Europe-wide review noted that apart from a few exceptions, virtually all national surveys report mean added sugar intake above 10% of energy [10]. The Feeding Infants and Toddlers Study (FITS) 2016 in the United States similarly found that a notable proportion of toddlers (particularly in low-income groups) exceeded added sugar consumption guidelines [33]. An earlier study assessing the diets of Polish toddlers aged 12–36 months reported that added sugar was present in the diets of 32.9% to 54.1% of children, depending on age group, indicating a high prevalence of added sugar consumption in early childhood [34]. Continuation of this trend suggests a need for stricter regulation and education around sugar-containing foods and beverages introduced during complementary feeding and carried into later childhood.

Simultaneously, fibre intake tended to be insufficient and was closely related to diet quality patterns. Only less than 5% of toddlers met the adequate intake for fibre in the United States, and European children also commonly under-consume fibre [10,33], mainly as a result of diets high in refined carbohydrates and low in whole grains.

### 4.2. Micronutrient Intake

Micronutrient intake analysis revealed a high prevalence of inadequacy in vitamin D, E, and calcium, alongside overconsumption of sodium.

However, our study only informed about the risk of inadequate nutrient intake and did not examine nutrient deficiencies, the literature indicates that vitamin D insufficiency affected the majority of children across all age strata, consistent with previous national and international observations [33,35,36,37]. Despite widespread recommendations for supplementation, adherence remains low [38]. In our study, the risk of inadequate vitamin D intake was common among children aged 13–72 months, indicating that this nutrient is one of the lowest in compliance with recommended intakes. The healthy or unhealthy diet patterns were not associated with decreased risk of inadequate vitamin D intake, indicating a universal need for supplementation. Vitamin D deficiency has been described as a pandemic, a public health issue that requires action [39].

Very low vitamin E intake was reported in Poland earlier [5]. The highest risk of inadequate intake of vitamin E was in the post-infancy period.

Calcium intakes were insufficient in a significant proportion of toddlers and preschoolers. The risk of inadequate calcium intake increased rapidly after infancy and was highly associated with a pro-healthy dietary pattern in both age groups. The risk of calcium deficiency is common among toddlers and preschoolers in Europe, especially after breastfeeding or formula use declines. In our study, in these groups, almost two-thirds of children were at risk of insufficient calcium intake, which is significantly higher than reported in Spain and the United States (≤10%) [33,40].

Observed high sodium intake was a consistent issue across Europe and the United States, reflecting the universal challenge faced by developed countries. Exposure to high sodium begins very early, as sodium consumption in the majority of infants exceeds the AI. A worldwide review and meta-analysis of studies estimated that 73% of children had high sodium intake, and this only slightly decreased between 1990 and 2021. Sodium consumption increased logarithmically with age from 0 to 18 years [41]. This analysis also highlighted the low potassium intake; however, in the PITNUTS 2024, intake below the AI was uncommon. Although potassium intake may be adequate, concurrent high sodium consumption can still disrupt electrolyte balance and lead to adverse health outcomes. Moreover, sodium intake is often underestimated because dietary assessment tools typically rely on standardized food items, which may not accurately reflect the higher salt content of homemade dishes.

### 4.3. Diet Quality Patterns and Nutrient Adequacy

Our analysis of diet quality scores (child-pHDS and child-nHDS) suggests that children’s adherence to healthy and unhealthy dietary patterns was partly related to their risk of nutrient inadequacies. In general, children with higher child-pHDS (i.e., diets rich in vegetables, fruits, whole grains, and other nutrient-dense foods) showed lower risk of excessive sugar consumption and some micronutrient shortfalls. In contrast, children aged 27–72 months scoring high on the child-nHDS were more likely to have insufficient intakes of ALA and fibre. Although the relations were not always clear. In younger children, the risk of inadequacies, which depended on diet quality levels, was more prominent than in the older group. However, the significant associations were revealed in both age groups in the logistic regression analysis after accounting for confounding factors. Among children aged 13–36 months, a healthy diet pattern was associated with a nearly five times lower risk of inadequate intake of EPA and DHA; however, no such association was found in the older group or in children with a high non-healthy diet pattern, regardless of their age. On the one side, this may suggest that positive dietary patterns tend to co-occur, i.e., a higher frequency of consumption of vegetables, nuts, fruits, cereal products, unsweetened dairy products, unprocessed animal-source foods, and recommended fats was associated with higher fish intake, which is the primary dietary source of EPA and DHA. Results from other cohorts indicate that healthy or prudent dietary patterns in early childhood include higher fish consumption. In UK and Canadian preschoolers, fish consumption is positively associated with health-conscious/prudent eating patterns, alongside vegetables, legumes, and fruits [42,43]. However, this does not explain why a high child-pHDS did not decrease the risk of inadequate EPA and DHA in older preschool toddlers. The possible answer can be a diversity of diets.

Surprisingly, a high child-nHDS had an opposite effect on the proportion of energy derived from ALA, depending on age. In children aged 13–36 months, a higher child-nHDS may indicate the consumption of processed foods containing plant oils, which increases the intake of ALA. However, by 37–72 months, the unhealthy pattern can be dominated by foods with negligible ALA, leading to a reversal of the association. This age-dependent shift may reflect both changes in the food supply and dietary displacement. This observation requires further analysis.

Overall, developed diet quality patterns are associated with the nutritional value of the diet and can be useful in predicting its nutritional value. Although some associations, such as the risk of inadequate calcium intake based on the child-pHDS, were observed in our study, there were inconsistencies across age groups and scores, illustrating that both healthy and unhealthy dietary practices tend to cluster and influence nutrient adequacy in complex ways. The utility of pattern-based approaches lies in their ability to capture the overall dietary context rather than single nutrient intakes, which may be particularly valuable for identifying children at risk of hidden deficiencies despite apparently sufficient energy intake. Further analyses and studies are needed to confirm these associations and to clarify age-related differences in how dietary patterns affect specific nutrient intakes. Nevertheless, our findings support the inclusion of dietary pattern analysis in nutritional monitoring and emphasize the importance of promoting diverse, balanced diets from the earliest years of life to improve nutrient adequacy and long-term health outcomes.

### 4.4. Public Health Implications

The PITNUTS 2024 data repeat several key findings from the 2016 edition [5]. In both studies, protein overconsumption, suboptimal fatty acid profiles, and high carbohydrate intake were widespread across all age groups. Nevertheless, the persistence of the risk of micronutrient deficiencies, particularly for vitamin D, suggests that education campaigns and supplementation programmes have not reached their intended goals. Moreover, the prevalence of overweight or risk of being overweight remains at a similar level, i.e., 17.9% and 14.7% in infants and 28.1% and 28.4% in preschoolers, in 2016 and 2024, respectively [5]. This underscores the urgency of strengthening the implementation of both dietary recommendations and food environment policies for young children. Observed in the PITNUTS 2024 study, nutrition-related risks have multiple public health implications. Excess of sodium in childhood contributes to higher blood pressure and tracks into later life [44]. An observed high sucrose intake increases the risk of dental caries and weight gain, irrespective of other dietary or macronutrient intakes [45,46]. We believe that to promote healthy nutrition choices, the labelling of salt and sugar content in foods should be more prominent, taking into account the level of adults’ health literacy. The high SFA contribution, inconsistent with WHO guidance for children, can be linked to adverse blood-lipid profiles [26], and requires replacing SFA with unsaturated fats, i.e., increasing fish consumption. The widespread risk of inadequate vitamin D and calcium after infancy has implications for bone health and justifies strengthening routine vitamin D prophylaxis and promoting calcium-rich foods or fortified alternatives, in line with recent Polish recommendations [38]. This may require revisiting current distribution strategies and improving adherence through healthcare provider engagement and public awareness campaigns.

The evidence on diet quality patterns in young children carries important implications for public health policy and caregiver education. Many countries lack dietary data for the youngest children [10], which can result in undiagnosed dietary problems. In our study, the dietary patterns of children in the post-infancy period were diverse, indicating that in many children, feeding practices deviated from the principles of proper food selection in daily dietary intake, resulting in inadequate energy and nutritional value. The high score of the child-pHDS was associated with a lower risk of inadequate intake of macronutrients and multiple micronutrients with frequent gaps in intake; however, only 14–16% of the diets of children aged 13–72 months adhered to that pattern. In our study, the high child-nHDS quality score had a lesser impact than child-pHDS on reducing risk of inadequate intake of nutrients. However, in reality, promoting only good or avoiding not-so-good dietary patterns is not possible. Both approaches have value when they are based on real consumption information monitoring, which allows to modify eating behaviour by setting goals [47]. Observed diversity in dietary patterns, with significant proportions of children receiving moderately healthy or unhealthy diets, indicates that many diets are not as nutritionally balanced as they could be. By monitoring these trends and implementing evidence-based interventions, such as educating parents and caregivers, and updating food policies and attitudes towards supplementation, it is possible to improve diet quality in early childhood. Such improvements are likely to yield better nutrient adequacy and healthier growth. Finally, the data support the need for revisions to dietary guidelines that emphasize not only nutrient adequacy but also the balance and diversity of food intake, with particular attention to reducing excessive consumption of protein, saturated fat, and added sugar.

### 4.5. Strengths and Limitations

The strengths of our study include its ability to be benchmarked against other national surveys, thanks to the collection of comparable intake data. This allows us to place our findings in an international context and confirm that the trends observed are part of a wider pattern. Additionally, the wide age range (5–72 months) and large, stratified, and nationally representative sample size provide a comprehensive overview that overlaps with numerous studies from Europe and the United States, facilitating meaningful comparison of diet quality across countries. The use of standardized, validated qualitative and quantitative dietary assessment methods enhanced comparability. The PITNUTS 2024 study utilized predefined dietary patterns, informed by diet-nutrition knowledge. In contrast, the earlier study, PITNUTS 2016, characterized dietary patterns based solely on available diet data and identified three clusters of dietary patterns associated with overweight and obesity [6].

The study also has limitations. Its cross-sectional design prevents the determination of causality between dietary patterns and nutritional outcomes. Dietary intake data rely on parent-reported intake, which can be subject to recall bias and under-reporting of unhealthy foods or over-reporting of healthy foods. However, quantitative dietary data based on a 3-day food record decreases the risk of recall bias. High refusal-to-participate rate and exclusion of specific subgroups (e.g., infants with minimal complementary feeding) could introduce selection bias. Additionally, seasonality may have played an important role in changes in dietary intake. Finally, the study lacks laboratory measures and cannot confirm nutrient deficiencies clinically; thus, it focused only on the risk of inadequate nutrient intake.

## 5. Conclusions

Findings from the PITNUTS 2024 study provides an updated and comprehensive assessment of diet quality and nutrient adequacy among nationally representative sample of Polish children aged 5–72 months. It has been shown that the developed diet quality scores can be useful for identifying children at risk of inadequate nutrient intake which can help in monitoring children’s nutrition over the years.

The most consistent problems across age groups were inadequate vitamin D and LCPUFA intake as well as excessive sodium intake, occurring in nearly all children after infancy. Most children also showed saturated fat intake above recommended level. Protein intake exceeding the acceptable macronutrient distribution range, particularly among toddlers. Insufficient calcium and fibre intake, and sucrose intake exceeding 10% of energy was observed in about half of toddlers and most preschoolers.

Priority actions should include implementing strategies to prevent insufficient vitamin D intake, providing effective dietary guidance to reduce free sugars, salt, and SFA while maintaining adequate total fat, and improving calcium intake through efficacious promotion of healthy choices, interventions in primary care, food labelling, and leveraging fortified foods. These actions are consistent with WHO nutrition guidance for children and directly address the largest gaps observed in the PITNUTS 2024.

## Figures and Tables

**Table 1:** Characteristics of the study sample by children’s age groups (*n* = 940).

Variables	Children Aged 5–12 Months						Children Aged 13–36 Months		Children Aged 37–72 Months	
	Total Sample		Breastfed		Non-Breastfed					
	*n*	%	*n*	%	*n*	%	*n*	%	*n*	%
Sample size	240/236/225 ^2^		130/128/123 ^2^		110/108/102 ^2^		400/390/388 ^2^		300/290/296 ^2^	
Sex										
male	108	45.0	61	46.9	47	42.7	188	47.0	138	46.0
female	132	55.0	69	53.1	63	57.3	212	53.0	162	54.0
Age (months) ^1^	9.0 (7.0–11.0)		9.0 (7.0–11.0)		9.0 (6.0–11.0)		24.5 (16.0–30.0)		55.0 (45.0–64.0)	
Type of municipality										
rural	72	30.0	42	32.3	30	27.3	124	31.0	93	31.0
urban–rural	56	23.3	30	23.1	26	23.6	94	23.5	71	23.7
urban ≤100,000 inhabitants	48	20.0	21	16.2	27	24.5	78	19.5	58	19.3
urban >100,000 inhabitants	64	26.7	37	28.5	27	24.5	104	26.0	78	26.0
Economic situation of household										
poor	3	1.3	2	1.5	1	0.9	2	0.5	2	0.7
average	70	29.2	42	32.3	28	25.5	117	29.3	93	31.0
good	143	59.6	74	56.9	69	62.7	237	59.3	164	54.7
very good	24	10.0	12	9.2	12	10.9	44	11.0	41	13.7
Maternal education level										
primary/lower secondary	5	2.1	1	0.8	4	3.6	9	2.3	7	2.3
basic vocational	26	10.8	13	10.0	13	11.8	35	8.8	32	10.7
secondary	114	47.5	58	44.6	56	50.9	188	47.0	149	49.7
higher	95	39.6	58	44.6	37	33.6	168	42.0	112	37.3
Paternal education level										
primary/lower secondary	4	1.7	1	0.8	3	2.8	8	2.1	10	3.4
basic vocational	45	19.1	14	10.9	31	28.7	71	18.2	61	21.0
secondary	116	49.2	70	54.7	46	42.6	188	48.2	144	49.7
higher	71	30.1	43	33.6	28	25.9	123	31.5	75	25.9
Parent-proxy reporter										
father	10	4.2	7	5.4	3	2.7	29	7.3	22	7.3
mother	230	95.8	123	94.6	107	97.3	371	92.8	278	92.7
Nutrition knowledge of parent-proxy reporter										
insufficient	0	0.0	0	0.0	0	0.0	1	0.3	0	0.0
sufficient	14	5.8	7	5.4	7	6.4	30	7.5	25	8.3
good	165	68.8	91	70.0	74	67.3	282	70.5	214	71.3
very good	61	25.4	32	24.6	29	26.4	87	21.8	61	20.3
Physical activity level compared to peers										
similar	206	85.8	112	86.2	94	85.5	323	80.8	231	77.0
lower	14	5.8	8	6.2	6	5.5	9	2.3	15	5.0
higher	20	8.3	10	7.7	10	9.1	68	17.0	54	18.0
BMI-for-age z-score ^3^										
underweight	36	16.0	19	15.4	17	16.7	35	9.0	25	8.4
normal weight	178	79.1	101	82.1	77	75.5	315	81.2	214	72.3
overweight	11	4.9	3	2.4	8	7.8	27	7.0	39	13.2
obesity	0	0.0	0	0.0	0	0.0	11	2.8	18	6.1

^1^ median value with lower and upper quartiles (Q1–Q3); ^2^ lower sample size for the paternal education level due to the lack of data and for BMI-for-age z-score due to risk of error in anthropometric measurements; ^3^ BMI-for-age z-scores—the age-specific Body Mass Index calculated using measured height and weight and assessed in categories according to the WHO standards.

**Table 2:** Median values (Me), lower and upper quartile ranges (Q1–Q3), and quartile coefficient of variation (CV_Q_) of daily energy and nutrient intake by children’s age groups.

Variables	Children Aged 5–12 Months Non-Breastfed (*n* = 110)			Children Aged 13–36 Months (*n* = 400)			Children Aged 37–72 Months (*n* = 300)			*p*
	Me	Q1–Q3	CV_Q_ (%)	Me	Q1–Q3	CV_Q_ (%)	Me	Q1–Q3	CV_Q_ (%)	
Energy (kcal)	740.4	615.8–854.6	32.3	1008.6	804.2–1220.4	41.3	1257.9	1066.5–1462.4	31.5	<0.001
Protein										
Protein (g)	19.4	14.7–25.4	55.2	38.5	28.4–47.8	50.4	50.3	41.7–59.3	35.0	<0.001
Protein (%E)	10.5	9.3–12.2	28.2	15.5	13.4–17.2	25.0	15.8	14.2–17.3	19.3	<0.001
Animal protein (g)	14.3	10.4–18.7	58.5	25.7	18.0–33.7	61.2	33.1	27.1–41.7	44.0	<0.001
Plant protein (g)	4.1	2.3–7.8	133.7	11.3	8.6–14.7	54.3	15.7	13.0–18.3	33.6	<0.001
Fats										
Fat (g)	25.5	20.5–30.8	40.4	34.1	24.5–41.0	48.5	41.4	34.1–50.5	39.6	<0.001
Fat (%E)	31.6	27.9–35.5	24.3	28.6	25.3–32.5	25.2	29.6	26.3–33.7	25.0	<0.001
SFA (g)	10.5	8.1–12.8	44.8	12.9	8.8–17.1	63.8	17.0	13.8–21.2	43.3	<0.001
SFA (%E)	12.8	10.4–14.9	35.5	11.5	9.5–13.4	33.9	12.2	10.7–14.0	27.1	<0.001
MUFA (g)	9.4	7.4–11.4	42.6	11.6	7.6–15.2	66.1	15.6	12.8–20.1	46.9	<0.001
PUFA (g)	4.2	3.5–5.1	38.0	4.0	3.0–5.4	58.3	4.9	4.0–6.5	51.8	<0.001
LA (g)	3.4	2.8–4.0	33.8	3.2	2.4–4.1	53.9	3.8	3.1–5.1	53.2	<0.001
LA (%E)	4.2	3.6–4.8	30.2	2.8	2.4–3.5	37.2	2.8	2.4–3.3	34.5	<0.001
ALA (g)	0.6	0.5–0.7	35.3	0.6	0.4–0.9	73.9	0.9	0.6–1.1	63.4	<0.001
ALA (%E)	0.8	0.7–0.9	31.6	0.6	0.4–0.8	55.0	0.6	0.5–0.8	54.8	<0.001
EPA (mg)	3.2	0.0–7.8	242.7	3.5	0.7–11.3	300.4	5.4	2.2–14.7	230.9	<0.001
DHA (mg)	17.2	4.0–49.6	264.8	37.3	18.4–65.6	126.7	36.0	18.7–59.8	114.2	<0.001
EPA + DHA (mg)	24.6	6.3–58.8	213.7	43.8	22.4–73.9	117.4	40.2	22.3–70.1	119.0	<0.001
Carbohydrates										
Carbohydrates (g)	109.1	87.7–133.4	41.9	142.0	114.2–174.6	42.5	172.0	147.9–206.9	34.3	<0.001
Carbohydrates (%E)	54.8	51.3–58.7	13.6	53.2	49.1–57.9	16.5	52.7	48.7–56.4	14.7	0.003
Starch (g)	23.7	11.7–44.7	139.1	60.4	44.6–80.0	58.7	85.4	69.4–101.8	37.9	<0.001
Lactose (g)	41.0	26.7–55.3	69.7	14.0	7.6–22.6	107.2	11.8	8.0–16.9	74.6	<0.001
Sucrose (g)	11.3	5.4–19.3	123.2	25.5	16.6–34.2	69.2	35.5	25.3–45.2	56.2	<0.001
Sucrose (%E)	6.0	3.5–8.5	83.8	9.9	7.2–13.1	59.3	11.0	8.7–13.8	45.9	<0.001
Fibre (g)	8.3	6.6–10.3	43.8	9.9	8.0–12.4	45.2	10.8	8.6–13.9	48.9	<0.001
Minerals										
Sodium (mg)	362.7	202.7–781.1	159.5	1453.3	1081.8–1902.7	56.5	2060.2	1712.6–2441.9	35.4	<0.001
Potassium (mg)	1211.9	972.8–1536.1	46.5	1653.5	1358.5–2128.1	46.5	1982.0	1657.5–2412.4	38.1	<0.001
Calcium (mg)	528.3	414.5–634.7	41.7	534.5	369.3–684.6	59.0	550.8	444.4–708.7	48.0	0.021
Phosphorus (mg)	441.1	357.3–569.6	48.1	697.9	545.7–845.7	43.0	809.8	670.4–968.4	36.8	<0.001
Magnesium (mg)	88.6	67.8–124.2	63.7	147.0	115.9–184.9	47.0	184.8	149.8–223.1	39.7	<0.001
Iron (mg)	7.4	5.6–9.1	47.4	6.2	4.7–7.7	47.0	6.2	5.0–7.6	42.6	<0.001
Zinc (mg)	4.9	4.1–5.9	37.8	5.1	4.1–6.4	46.7	5.7	4.6–6.9	40.2	<0.001
Copper (mg)	0.4	0.4–0.5	40.6	0.5	0.4–0.7	54.3	0.7	0.5–0.8	44.3	<0.001
Manganese (mg)	0.7	0.4–1.2	110.6	1.6	1.1–2.2	69.5	2.3	1.8–2.9	47.7	<0.001
Iodine (μg)	102.4	85.2–118.2	32.3	87.4	62.3–114.3	59.5	85.7	66.5–103.0	42.6	<0.001
Vitamins										
Vitamin A (μg)	883.0	625.4–1179.2	62.7	780.8	553.1–1088.1	68.5	760.5	498.4–992.1	64.9	0.007
Retinol (μg)	23.8	4.2–80.8	321.7	192.6	118.0–272.5	80.2	262.9	202.1–337.3	51.4	<0.001
β-carotene (μg)	2617.1	1469.3–4019.1	97.4	2694.9	1716.3–4515.4	103.9	2587.9	1526.4–4229.4	104.4	0.406
Vitamin D (μg)	8.6	6.2–10.9	54.7	2.4	1.2–7.1	243.2	1.3	0.9–1.9	79.1	<0.001
Vitamin E (mg)	8.2	6.6–9.8	39.0	4.9	3.5–6.5	60.0	4.9	3.9–6.5	53.8	<0.001
Vitamin B_1_ (mg)	0.6	0.5–0.7	44.2	0.6	0.5–0.8	51.5	0.7	0.6–0.9	40.6	<0.001
Vitamin B_2_ (mg)	0.9	0.8–1.1	38.2	1.1	0.9–1.5	53.7	1.2	1.0–1.5	42.3	<0.001
Niacin (mg)	6.1	4.8–7.3	42.3	7.7	5.6–10.1	58.8	9.8	7.4–12.7	54.3	<0.001
Vitamin B_6_ (mg)	0.7	0.5–0.9	50.2	1.0	0.8–1.3	49.1	1.2	1.0–1.4	37.7	<0.001
Folates (μg)	183.4	153.7–211.4	31.5	158.8	122.8–191.9	43.5	163.6	131.1–207.2	46.5	<0.001
Vitamin B_12_ (μg)	1.4	1.1–1.9	53.3	2.1	1.6–2.8	57.8	2.2	1.7–2.8	47.5	<0.001
Vitamin C (mg)	82.4	67.4–96.6	35.3	63.7	43.5–87.8	69.5	53.0	36.8–75.8	73.6	<0.001

CV_Q_—quartile coefficient of variation ((Q3 − Q1)/Me × 100%), *p*—significance level of Kruskal–Wallis test with FDR correction, E—daily energy intake, SFA—saturated fatty acids, MUFA—monounsaturated fatty acids, PUFA—polyunsaturated fatty acids, LA—linoleic acid, ALA—α-linolenic acid, EPA—eicosapentaenoic acid, DHA—docosahexaenoic acid.

**Table 3:** Percentage of children (%) at risk of inadequate nutrient intake by children’s age groups.

Variables	Criterion	Children Aged 5–12 Months Non-Breastfed (*n* = 110)		Children Aged 13–36 Months (*n* = 400)		Children Aged 37–72 Months (*n* = 300)		*p*
		n	%	n	%	n	%	
Protein								
Protein (g)	<AI or EAR ^3^	14	12.7	11	2.8	0	0.0	<0.001
Protein (%E)	<AMDR	0	0.0	3	0.8	0	0.0	0.156
Protein (%E)	>AMDR	10	9.1	117	29.3	19	6.3	<0.001
Fats								
Fat (%E) ^1^	<AMDR	82	100.0	340	85.0	6	2.0	<0.001
Fat (%E) ^1^	>AMDR	0	0.0	22	5.5	49	16.3	0.003
SFA (%E)	≥10%	87	79.1	263	65.8	253	84.3	<0.001
LA (%E)	<AI	49	44.5	356	89.0	283	94.3	<0.001
ALA (%E)	<AI	11	10.0	154	38.5	90	30.0	<0.001
EPA + DHA (mg) ^1^	<AI	76	92.7	349	87.3	282	94.0	0.011
Carbohydrates								
Carbohydrates (%E)	<AMDR	1	0.9	41	10.3	30	10.0	0.260
Carbohydrates (%E)	>AMDR	69	62.7	17	4.3	5	1.7	<0.001
Sucrose (%E)	≥10%	20	18.2	196	49.0	184	61.3	<0.001
Fibre (g) ^2^	<AI	NA	NA	205	51.3	227	75.7	<0.001
Minerals								
Sodium (mg)	>AI	73	66.4	349	87.3	297	99.0	<0.001
Potassium (mg)	<AI	2	1.8	10	2.5	6	2.0	0.864
Calcium (mg)	<AI or EAR ^3^	4	3.6	176	44.0	256	85.3	<0.001
Phosphorus (mg)	<AI or EAR ^3^	5	4.5	34	8.5	3	1.0	<0.001
Magnesium (mg)	<AI or EAR ^3^	13	11.8	15	3.8	12	4.0	0.003
Iron (mg)	<AI or EAR ^4^	25	22.7	18	4.5	21	7.0	<0.001
Zinc (mg)	<AI or EAR ^4^	3	2.7	18	4.5	31	10.3	0.003
Copper (mg)	<AI or EAR ^3^	7	6.4	28	7.0	1	0.3	<0.001
Manganese (mg)	<AI	27	24.5	117	29.3	43	14.3	<0.001
Iodine (μg)	<AI or EAR ^3^	85	77.3	119	29.8	66	22.0	<0.001
Vitamins								
Vitamin A (μg)	<AI or EAR ^3^	1	0.9	6	1.5	8	2.7	0.398
Vitamin D (μg)	<AI	69	62.7	382	95.5	300	100.0	<0.001
Vitamin E (mg)	<AI	13	11.8	270	67.5	208	69.3	<0.001
Vitamin B_1_ (mg)	<AI or EAR ^3^	1	0.9	58	14.5	43	14.3	<0.001
Vitamin B_2_ (mg)	<AI or EAR ^3^	1	0.9	13	3.3	1	0.3	0.016
Niacin (mg)	<AI or EAR ^3^	20	18.2	77	19.3	35	11.7	0.028
Vitamin B_6_ (mg)	<AI or EAR ^3^	2	1.8	8	2.0	1	0.3	0.174
Folates (μg)	<AI or EAR ^3^	0	0.0	90	22.5	139	46.3	<0.001
Vitamin B_12_ (μg)	<AI or EAR ^3^	2	1.8	17	4.3	7	2.3	0.260
Vitamin C (mg)	<AI or EAR ^3^	1	0.9	47	11.8	91	30.3	<0.001

*p*—significance level of chi^2^ test with FDR correction, E—daily energy intake, SFA—saturated fatty acids, LA—linoleic acid, ALA—α-linolenic acid, EPA—eicosapentaenoic acid, DHA—docosahexaenoic acid, AI—adequate intake, EAR—estimated average requirement, AMDR—acceptable macronutrient distribution range, ^1^ Polish dietary recommendations for fat or EPA + DHA are available for children aged > 6 months (*n* = 82), ^2^ Polish dietary recommendations for fibre are available for children aged > 12 months, ^3^ depending on the child age: <AI for children aged 5–12 months and <EAR for children aged 13–72 months, ^4^ <AI for children aged 5–6 months and <EAR for children aged 7–72 months, NA—not available.

**Table 4:** Descriptive statistics and percentage distribution of children (%) for diet quality scores by children’s age groups.

Variables	Children Aged 13–36 Months (*n* = 400)		Children Aged 37–72 Months (*n* = 300)	
	*n*	%	*n*	%
Child-pHDS				
Me, Q1–Q3 (in points)	3 (2–5)		3 (2–5)	
Child-pHDS categories				
Low (0–2 points)	155	38.8	115	38.3
Moderate (3–5 points)	180	45.0	142	47.3
High (6–7 points)	65	16.2	43	14.4
Child-nHDS				
Me, Q1–Q3 (in points)	4 (1–6)		4 (3–6)	
Child-nHDS categories				
Low (0–3 points)	174	43.5	126	42.0
Moderate (4–7 points)	169	42.3	133	44.3
High (8–10 points)	57	14.2	41	13.7

Child-pHDS—Children’s pro-Healthy Diet Score developed for children aged 13–72 months (low: 0–2 points, moderate: 3–5 points, high: 6–7 points), Child-nHDS—Children’s non-Healthy Diet Score developed for children aged 13–72 months (low: 0–3 points, moderate: 4–7 points, high: 8–10 points), Me—median values, Q1—lower quartile, Q3—upper quartile.

**Table 5:** Adjusted odds ratios (OR_adj_, 95% CI) for the risk of inadequate nutrient intake according to the dietary recommendations by the diet quality levels in children aged 13–72 months.

Variables	Criterion	Children Aged 13–36 Months				Children Aged 37–72 Months			
		Child-pHDS High vs. Low (Ref.)		Child-nHDS High vs. Low (Ref.)		Child-pHDS High vs. Low (Ref.)		Child-nHDS High vs. Low (Ref.)	
		OR_adj_	95% CI	OR_adj_	95% CI	OR_adj_	95% CI	OR_adj_	95% CI
Protein									
Protein (g)	<AI or EAR ^1^	NA	NA	NA	NA	NA	NA	NA	NA
Protein (%E)	<AMDR	NA	NA	NA	NA	NA	NA	NA	NA
Protein (%E)	>AMDR	2.73 *	1.09–6.83	1.41	0.54–3.68	0.36	0.04–3.20	0.61	0.12–3.12
Fats									
Fat (%E)	<AMDR	0.42	0.12–1.49	0.78	0.22–2.78	1.49	0.10–22.40	NA	NA
Fat (%E)	>AMDR	0.44	0.04–4.46	0.00	0.00–4.91	0.82	0.25–2.67	1.28	0.45–3.69
SFA (%E)	≥10%	1.95	0.94–4.04	1.11	0.51–2.42	0.54	0.20–1.43	0.85	0.29–2.54
LA (%E)	<AI	0.74	0.26–2.07	0.54	0.19–1.58	0.73	0.13–4.11	5.95	0.41–86.16
ALA (%E)	<AI	1.05	0.54–2.07	0.41 *	0.19–0.91	1.00	0.42–2.37	2.55 *	1.04–6.28
EPA + DHA (mg)	<AI	0.22 **	0.07–0.64	0.85	0.28–2.55	1.21	0.18–8.31	NA	NA
Carbohydrates									
Carbohydrates (%E)	<AMDR	3.14 *	1.05–9.35	1.29	0.44–3.74	1.04	0.27–3.97	2.75	0.87–8.74
Carbohydrates (%E)	>AMDR	NA	NA	0.35	0.04–3.02	1.37	0.09–20.31	NA	NA
Sucrose (%E)	≥10%	0.57	0.28–1.16	1.49	0.72–3.10	0.41 *	0.18–0.92	1.58	0.69–3.61
Fibre (g)	<AI	0.39 **	0.20–0.79	0.92	0.45–1.87	0.44	0.18–1.06	3.44 *	1.03–11.55
Minerals									
Sodium (mg)	>AI	3.23	0.59–17.70	0.73	0.17–3.12	NA	NA	NA	NA
Potassium (mg)	<AI	0.46	0.03–7.70	2.44	0.05–111.03	NA	NA	NA	NA
Calcium (mg)	<AI or EAR ^1^	0.45 *	0.22–0.91	0.73	0.36–1.51	0.16 **	0.05–0.52	1.14	0.37–3.49
Phosphorus (mg)	<AI or EAR ^1^	0.29	0.05–1.58	0.41	0.03–5.55	NA	NA	NA	NA
Magnesium (mg)	<AI or EAR ^1^	0.27	0.02–3.60	2.20	0.06–79.78	NA	NA	10.35 *	1.31–81.93
Iron (mg)	<AI or EAR ^2^	0.80	0.12–5.44	0.63	0.04–11.13	0.23	0.02–2.29	2.69	0.60–12.04
Zinc (mg)	<AI or EAR ^2^	NA	NA	NA	NA	0.30	0.06–1.66	2.18	0.59–8.08
Copper (mg)	<AI or EAR ^1^	0.16	0.02–1.66	1.06	0.17–6.70	NA	NA	NA	NA
Manganese (mg)	<AI	0.47	0.20–1.10	1.39	0.59–3.25	0.92	0.21–4.08	1.50	0.50–4.54
Iodine (μg)	<AI or EAR ^1^	0.60	0.29–1.27	0.96	0.43–2.16	0.58	0.22–1.55	0.93	0.34–2.58
Vitamins									
Vitamin A (μg)	<AI or EAR ^1^	NA	NA	NA	NA	0.34	0.02–5.35	NA	NA
Vitamin D (μg)	<AI	1.15	0.09–15.13	0.28	0.05–1.66	NA	NA	NA	NA
Vitamin E (mg)	<AI	0.53	0.26–1.09	0.57	0.27–1.22	0.55	0.22–1.33	1.60	0.61–4.20
Vitamin B_1_ (mg)	<AI or EAR ^1^	0.20 *	0.04–0.90	0.28	0.06–1.40	0.53	0.15–1.95	2.21	0.62–7.80
Vitamin B_2_ (mg)	<AI or EAR ^1^	NA	NA	NA	NA	NA	NA	NA	NA
Niacin (mg)	<AI or EAR ^1^	0.19 **	0.06–0.59	1.34	0.52–3.45	0.99	0.29–3.43	1.20	0.30–4.78
Vitamin B_6_ (mg)	<AI or EAR ^1^	NA	NA	NA	NA	NA	NA	NA	NA
Folates (μg)	<AI or EAR ^1^	0.24 **	0.09–0.62	1.06	0.43–2.58	0.67	0.30–1.48	1.38	0.59–3.21
Vitamin B_12_ (μg)	<AI or EAR ^1^	NA	NA	NA	NA	NA	NA	NA	NA
Vitamin C (mg)	<AI or EAR ^1^	0.24 *	0.06–0.89	0.68	0.20–2.29	0.57	0.23–1.37	2.31	0.97–5.51

Child-pHDS—Children’s pro-Healthy Diet Score developed for children aged 13–72 months (low: 0–2 points, high: 6–7 points), Child-nHDS—Children’s non-Healthy Diet Score developed for children aged 13–72 months (low: 0–3 points, high: 8–10 points), OR_adj_—odds ratio adjusted for 7 variables: sex, age (months), BMI-for-age z-score, education levels of mother and father, BMIs of mother and father; CI—confidence interval, E—daily energy intake, SFA—saturated fatty acids, LA—linoleic acid, ALA—α-linolenic acid, EPA—eicosapentaenoic acid, DHA—docosahexaenoic acid, AI—adequate intake, EAR—estimated average requirement, AMDR—acceptable macronutrient distribution range, ^1^ depending on the child age: <AI for children aged 5–12 months and <EAR for children aged 13–72 months, ^2^ <AI for children aged 5–6 months and <EAR for children aged 7–72 months; * *p* < 0.05, ** *p* < 0.01 (significance level of Wald’s test); NA—odds ratios are not available due to the zero values in contingency tables.

## Data Availability

The raw data supporting the conclusions of this article will be made available from the corresponding author upon reasonable request.

## Supplementary Materials

The following supporting information can be downloaded at: https://www.mdpi.com/article/10.3390/nu17213364/s1, Table S1: Territorial representation of the drawn sample in voivodeships and municipalities of different types; Table S2: Components of the Children’s pro-Healthy Diet Score and their scoring; Table S3: Components of the Children’s non-Healthy Diet Score and their scoring; Table S4: Dietary recommendations by children’s age groups; Table S5: Median values (Me) with lower and upper quartile ranges (Q1–Q3) of daily energy and nutrient intake by the diet quality levels in children aged 13–36 months (*n* = 400); Table S6: Median values (Me) with lower and upper quartile ranges (Q1–Q3) of daily energy and nutrient intake by the diet quality levels in children aged 37–72 months (*n* = 300); Table S7: Percentage of children (%) at risk of inadequate nutrient intake according to the dietary recommendations by the diet quality levels in children aged 13–36 months (*n* = 400); Table S8: Percentage of children (%) at risk of inadequate nutrient intake according to the dietary recommendations by the diet quality levels in children aged 37–72 months (*n* = 300); Table S9: Crude odds ratios (ORs, 95% CI) for the risk of inadequate nutrient intake according to the dietary recommendations by the diet quality levels in children aged 13–72 months.

## Abbreviations

The following abbreviations are used in this manuscript:

AI	Adequate Intake
ALA	α-Linolenic Acid
AMDR	Acceptable Macronutrient Distribution Range
BMI	Body Mass Index
CAPI	Computer-Assisted Personal Interview
child-nHDS	Children’s non-Healthy Diet Score
child-pHDS	Children’s pro-Healthy Diet Score
CVQ	Quartile Coefficient of Variation
DHA	Docosahexaenoic Acid
E	Daily Energy Intake
EAR	Estimated Average Requirement
EPA	Eicosapentaenoic Acid
ESPGHAN	European Society for Pediatric Gastroenterology, Hepatology and Nutrition
FFQ	Food Frequency Questionnaire
LA	Linoleic Acid
LCPUFA	Long-Chain Polyunsaturated Fatty Acids
MUFA	Monounsaturated Fatty Acids
PITNUTS	Polish Infant and Toddler Nutrition Study
PUFA	Polyunsaturated Fatty Acids
SFA	Saturated Fatty Acids
WHO	World Health Organization
