# Supplementary material for: Diet Quality and Nutrient Adequacy Among Polish Children: Findings from the PITNUTS 2024 Study
Source: Nutrients. 2025 Oct 26;17(21):3364. doi: 10.3390/nu17213364 (PMC12611091; doi:10.3390/nu17213364)
Supplement: Supplementary file 1 [file nutrients-17-03364-s001.zip › nutrients-3921895-supplementary.pdf]

Supplementary materials

Table S1. Territorial representation of the drawn sample in voivodships and municipalities of different types

Voivodeship	Overall (n = 1000)	Rural (n = 310)	Urban-rural (n = 235)	Urban ≤100,000 inhabitants (n = 195)	Urban >100,000 inhabitants (n = 260)
	n (%)	%	%	%	%
Dolnośląskie	80 (8.0)	6.5	8.5	10.3	7.7
Kujawsko-Pomorskie	50 (5.0)	6.5	4.3	5.1	3.8
Lubelskie	50 (5.0)	6.5	4.3	5.1	3.8
Lubuskie	30 (3.0)	3.2	4.3	0.0	3.8
Łódzkie	50 (5.0)	6.5	4.3	5.1	3.8
Małopolskie	100 (10.0)	12.9	12.8	5.1	7.7
Mazowieckie	160 (16.0)	14.5	10.6	15.4	23.1
Opolskie	20 (2.0)	3.2	4.3	0.0	0.0
Podkarpackie	50 (5.0)	6.5	4.3	5.1	3.8
Podlaskie	40 (4.0)	3.2	4.3	5.1	3.8
Pomorskie	70 (7.0)	4.8	4.3	12.8	7.7
Śląskie	110 (11.0)	6.5	4.3	15.4	19.2
Świętokrzyskie	20 (2.0)	3.2	4.3	0.0	0.0
Warmińsko-Mazurskie	40 (4.0)	3.2	4.3	5.1	3.8
Wielkopolskie	90 (9.0)	9.7	17.0	5.1	3.8
Zachodniopomorskie	40 (4.0)	3.2	4.3	5.1	3.8

Table S2. Components of the Children's pro-Healthy Diet Score and their scoring

No.	Components of the child-pHDS	Median daily consumption frequency [times/day] of food groups[1]		No. of FFQ item	Items of the FFQ included in the food group
		13–36 months (n = 400)	37–72 months (n = 300)		
1	Vegetables	1.28	1.28	37	Vegetables (fresh/frozen)
				38	Vegetables (e.g. boiled, including soups)
				40	Pickled vegetables
2	Pulses/nuts	0.06	0.12	41	Pulses and pulse-based dishes
				49	Nuts, seeds
3	Fruit	1.06	1.00	45	Fruit (fresh/frozen)
				46	Fruit mousse
4	Cereal products	0.60	0.77	2	Wholemeal bread
				5	Brown rice
				7	Whole grain pasta
				8	Groats
				11	Natural cereals (e.g. oat flakes)
5	Unsweetened dairy products	0.69	1.06	15	Whole milk
				16	Semi-skimmed milk
				17	Skimmed milk
				21	Natural yogurt, kefir, buttermilk
				23	Cottage cheese and other unsweetened cheeses
6	Meat/fish/eggs	1.28	1.34	28	White meat
				29	Red meat
				32	Fresh/frozen fish
				35	Seafood
				36	Eggs
7	Recommended fats	1.14	1.67	51	Butter
				54	Olive oil
				55	Vegetable oils

[1] one point is given for consumption of a food group exceeded the age-specific median [times/day]; the total score of the child-pHDS is calculated as the sum of points (range: 0-7 points)

Table S3. Components of the Children's non-Healthy Diet Score and their scoring

No.	Components of the child-nHDS	Median daily consumption frequency [times/day] of food groups[1]		No. of FFQ item	Items of the FFQ included in the food group
		13–36 months (n = 400)	37–72 months (n = 300)		
1	Sweetened breakfast cereals	0.06	0.14	9	Flavoured breakfast cereals
2	Fried flour-based dishes	0.14	0.14	13	Fried flour dishes (e.g. pancakes)
3	Fast food	0.06	0.12	14	Fast food (excluding French fries)
				43	French fries
4	Sweetened dairy products	0.63	0.85	18	Flavoured milk beverages (e.g. cocoa)
				19	Flavoured condensed milk
				22	Flavoured yogurt, buttermilk
				24	Flavoured homogenized cheeses
				25	Dairy desserts (e.g. pudding)
5	Processed meat	0.71	1.14	30	High-quality cold cuts
				31	Sausages, pates
6	Food concentrates/ salty seasonings	0.06	0.14	59	Salty condiments with vegetables (e.g. bouillon cubes)
				60	Instant soups and sauces
7	Sweet condiments	0.42	0.91	61	Sugar
				63	Fruit syrups
				64	Jam, marmalade
				65	Chocolate/chocolate-nut spreads
8	Sweets	0.57	0.83	66	Chocolate, chocolate bars
				67	Candies
				68	Sponge cookies, biscuits
				69	Other cookies
				70	Cakes and pastries
9	Salty snacks	0.06	0.06	72	Salty snacks
10	Sweetened beverages	0.34	0.79	74	Flavoured water (commercial)
				75	Sweetened tea
				77	Fruit compotes (sweetened)
				84	Sugar-sweetened soft drinks
				85	Diet soft drinks

[1] one point is given for consumption of a food group exceeded the age-specific median [times/day]; the total score of the child-nHDS is calculated as the sum of points (range: 0-10 points)

Table S4. Dietary recommendations by children's age groups

Variables	Children aged 5-12 months		Children aged 13-36 months	Children aged 37-72 months
	≤ 6 months	>6 months		
**Energy (kcal)**	550	700	1000	1400
**Protein**				
Protein (g)	9 (AI)	14 (AI)	12 (EAR)	16 (EAR)
Protein (%E)	5-15	5-15	5-15[1]/10-20[2]	10-20
**Fats**				
Fat (%E)	NA	40	35-40	20-35
SFA (%E)	<10	<10	<10	<10
LA (%E)	4 (AI)	4 (AI)	4 (AI)	4 (AI)
ALA (%E)	0.5 (AI)	0.5 (AI)	0.5 (AI)	0.5 (AI)
EPA + DHA (mg)	NA	100[1] (AI)	100[1]/250[2] (AI)	250 (AI)
**Carbohydrates**				
Carbohydrates (g)	60 (AI)	95 (AI)	100 (EAR)	100 (EAR)
Carbohydrates (%E)	40-45	45-55	45-65	45-65
Sucrose (%E)	<10	<10	<10	<10
Fibre (g)	NA	NA	10 (AI)	14 (AI)
**Minerals**				
Sodium (mg)	120 (AI)	370 (AI)	750 (AI)	1000 (AI)
Potassium (mg)	400 (AI)	750 (AI)	800 (AI)	1100 (AI)
Calcium (mg)	200 (AI)	260 (AI)	500 (EAR)	800 (EAR)
Phosphorus (mg)	150 (AI)	300 (AI)	380 (EAR)	410 (EAR)
Magnesium (mg)	30 (AI)	70 (AI)	65 (EAR)	110 (EAR)
Iron (mg)	0.3 (AI)	7 (EAR)	3 (EAR)	4 (EAR)
Zinc (mg)	2 (AI)	2.5 (EAR)	2.5 (EAR)	4 (EAR)
Copper (mg)	0.2 (AI)	0.3 (AI)	0.25 (EAR)	0.3 (EAR)
Manganese (mg)	0.003 (AI)	0.6 (AI)	1.2 (AI)	1.5 (AI)
Iodine (μg)	110 (AI)	130 (AI)	65 (EAR)	65 (EAR)
**Vitamins**				
Vitamin A (μg)	350 (AI)	350 (AI)	280 (EAR)	300 (EAR)
Vitamin D (μg)	10 (AI)	10 (AI)	15 (AI)	15 (AI)
Vitamin E (mg)	4 (AI)	5 (AI)	6 (AI)	6 (AI)
Vitamin B_1 (mg)	0.2 (AI)	0.3 (AI)	0.4 (EAR)	0.5 (EAR)
Vitamin B_2 (mg)	0.3 (AI)	0.4 (AI)	0.4 (EAR)	0.5 (EAR)
Niacin (mg)	2 (AI)	5 (AI)	5 (EAR)	6 (EAR)
Vitamin B_6 (mg)	0.1 (AI)	0.4 (AI)	0.4 (EAR)	0.5 (EAR)
Folates (μg)	65 (AI)	80 (AI)	120 (EAR)	160 (EAR)
Vitamin B_{12} (μg)	0.4 (AI)	0.5 (AI)	0.7 (EAR)	1.0 (EAR)
Vitamin C (mg)	20 (AI)	20 (AI)	30 (EAR)	40 (EAR)

E—daily energy intake, SFA—saturated fatty acids, MUFA—monounsaturated fatty acids, PUFA—polyunsaturated fatty acids, LA—linoleic acid, ALA—α-linolenic acid, EPA—eicosapentaenoic acid, DHA—docosahexaenoic acid, [1] for children ≤24 months, [2] for children >24 months, NA—not available.

Table S5. Median values (Me) with lower and upper quartile ranges (Q1-Q3) of daily energy and nutrient intake by the diet quality levels in children aged 13-36 months (n=400)

**Variables**	**Child-pHDS**							**Child-nHDS**						
	**Low (n=155)**		**Moderate (n=180)**		**High (n=65)**		**p**	**Low (n=174)**		**Moderate (n=169)**		**High (n=57)**		**p**
	**Me**	**Q1-Q3**	**Me**	**Q1-Q3**	**Me**	**Q1-Q3**		**Me**	**Q1-Q3**	**Me**	**Q1-Q3**	**Me**	**Q1-Q3**	
**Energy (kcal)**	885.6	683.5–1109.2	1055.4	858.7–1276.0	1113.6	999.1–1352.9	**<0.001**	905.8	733.0–1111.0	1090.1	847.8–1280.7	1037.6	918.5–1326.7	**<0.001**
**Protein**														
Protein (g)	30.8	22.8–42.2	40.0	31.9–50.6	45.4	38.7–55.5	**<0.001**	35.3	25.3–44.4	40.3	31.7–51.0	40.6	31.7–53.1	**<0.001**
Protein (%E)	14.7	12.4–16.7	15.6	13.8–17.4	16.4	14.4–17.7	**<0.001**	15.4	12.9–17.5	15.4	13.6–17.0	15.8	13.4–17.2	0.959
Animal protein (g)	19.5	14.6–28.0	26.4	21.5–35.5	31.4	25.0–41.2	**<0.001**	23.5	15.9–31.0	27.3	20.1–36.9	27.2	20.0–40.9	**<0.001**
Plant protein (g)	9.7	6.9–12.3	12.3	9.9–15.4	12.5	10.2–15.6	**<0.001**	10.0	7.6–13.1	12.2	9.4–15.6	12.5	10.3–14.8	**<0.001**
**Fats**														
Fat (g)	26.9	19.8–36.5	35.6	27.6–42.7	39.0	30.7–46.7	**<0.001**	28.2	20.9–38.7	36.1	27.0–42.4	35.9	28.5–44.3	**<0.001**
Fat (%E)	27.3	24.0–31.6	29.4	26.6–32.9	30.0	25.9–33.0	**0.001**	27.9	24.3–32.3	29.0	25.9–32.3	30.6	26.9–33.7	**0.031**
SFA (g)	9.5	6.9–15.3	13.8	10.5–17.5	15.9	10.8–19.6	**<0.001**	10.8	7.2–15.6	14.3	10.1–17.5	13.6	10.9–18.9	**<0.001**
SFA (%E)	10.6	8.0–12.9	11.6	9.9–13.6	12.2	9.9–13.4	**<0.001**	10.8	8.5–12.8	11.8	9.8–13.7	12.0	9.8–13.8	**0.027**
MUFA (g)	9.2	5.1–13.5	12.3	9.3–15.4	15.2	10.1–17.2	**<0.001**	9.1	5.9–13.9	12.1	9.3–15.8	13.9	10.7–17.4	**<0.001**
PUFA (g)	3.7	2.6–4.5	4.3	3.4–5.5	4.4	3.4–6.3	**<0.001**	3.8	2.8–4.7	4.2	3.2–5.6	4.5	3.4–6.4	**<0.001**
LA (g)	2.8	2.1–3.6	3.3	2.6–4.3	3.6	2.8–4.8	**<0.001**	3.0	2.2–3.7	3.4	2.6–4.4	3.3	2.6–4.8	**<0.001**
LA (%E)	2.8	2.4–3.5	2.8	2.4–3.3	2.9	2.4–3.6	0.984	2.8	2.4–3.5	2.9	2.4–3.4	2.9	2.4–3.6	0.959
ALA (g)	0.5	0.4–0.7	0.6	0.5–0.9	0.7	0.5–1.0	**<0.001**	0.5	0.4–0.7	0.7	0.5–0.9	0.8	0.5–1.2	**<0.001**
ALA (%E)	0.6	0.4–0.7	0.6	0.4–0.8	0.6	0.5–0.8	0.879	0.5	0.4–0.7	0.6	0.4–0.8	0.7	0.5–0.9	**0.049**
EPA (mg)	2.1	0.1–7.0	4.4	1.1–13.7	7.9	3.0–21.8	**<0.001**	3.3	0.9–10.8	3.3	0.5–11.9	5.2	0.9–12.1	0.626
DHA (mg)	31.2	14.1–55.1	40.3	21.0–75.1	42.6	27.1–74.4	**<0.001**	37.0	16.9–67.6	40.0	21.7–65.8	31.5	15.4–58.8	0.582
EPA + DHA (mg)	34.0	16.7–62.0	45.7	23.2–85.0	53.3	33.5–94.4	**<0.001**	44.2	20.3–71.3	44.6	24.3–74.7	40.5	20.2–65.2	0.751
**Carbohydrates**														
Carbohydrates (g)	125.9	96.3–160.2	146.5	119.1–180.9	150.3	129.4–189.6	**<0.001**	127.9	103.8–160.2	149.8	122.3–183.5	146.8	131.7–181.0	**<0.001**
Carbohydrates (%E)	55.7	50.5–59.8	52.2	48.4–56.0	51.7	46.6–55.3	**<0.001**	54.1	49.1–59.1	52.6	50.0–57.5	52.9	46.9–55.7	0.265
Starch (g)	52.6	35.3–68.3	66.2	49.6–82.8	68.0	52.9–87.3	**<0.001**	53.1	37.8–70.0	64.8	47.4–84.1	68.7	53.1–84.7	**<0.001**
Lactose (g)	15.4	8.2–23.3	11.9	7.0–22.4	15.6	9.3–21.7	0.301	13.6	7.2–23.9	14.4	7.6–20.8	12.3	8.7–21.8	0.914
Sucrose (g)	22.9	15.3–31.2	27.3	16.5–35.8	27.7	18.7–38.1	**0.012**	20.8	13.4–28.3	28.5	17.9–39.0	29.1	22.3–39.1	**<0.001**
Sucrose (%E)	10.1	7.6–13.9	9.9	7.1–12.6	9.7	7.0–13.1	0.556	9.4	6.7–12.2	10.7	7.5–13.8	11.1	8.2–13.1	0.076
Fibre (g)	9.0	7.2–11.0	10.5	8.4–12.9	10.8	8.9–13.2	**<0.001**	9.6	7.7–12.3	10.3	8.1–12.4	10.0	7.9–12.5	0.819
**Minerals**														
Sodium (mg)	1246.8	783.5–1532.6	1602.3	1192.5–1950.8	1613.7	1269.7–2205.8	**<0.001**	1401.2	849.4–1844.9	1535.9	1182.6–1931.6	1408.7	1222.7–2205.8	**0.006**
Potassium (mg)	1503.6	1243.3–1780.5	1776.9	1452.6–2231.7	2040.0	1510.9–2501.8	**<0.001**	1558.4	1318.2–2028.6	1702.7	1430.0–2171.6	1795.9	1442.4–2362.9	**0.031**
Calcium (mg)	504.2	339.6–643.3	535.7	372.2–705.8	604.3	468.2–748.1	**0.009**	505.0	340.5–687.2	551.3	385.6–674.7	538.0	384.1–728.3	0.332

Variables	Child-pHDS							Child-nHDS						
	Low (n=155)		Moderate (n=180)		High (n=65)		p	Low (n=174)		Moderate (n=169)		High (n=57)		p
	Me	Q1-Q3	Me	Q1-Q3	Me	Q1-Q3		Me	Q1-Q3	Me	Q1-Q3	Me	Q1-Q3	
Phosphorus (mg)	618.1	478.4–752.1	723.3	587.2–892.4	796.0	657.5–986.5	**<0.001**	662.9	502.7–780.8	723.5	566.6–885.5	716.8	577.8–948.5	**0.010**
Magnesium (mg)	130.7	104.8–154.8	157.3	128.6–192.8	165.7	133.1–218.5	**<0.001**	137.0	107.6–172.0	155.4	123.3–186.9	154.5	131.0–209.2	**0.006**
Iron (mg)	5.8	4.4–7.4	6.4	5.0–8.0	6.6	5.6–7.6	**0.015**	6.3	4.7–7.9	6.2	4.9–7.6	6.2	4.8–7.2	0.841
Zinc (mg)	4.6	3.6–5.8	5.3	4.4–6.6	5.4	4.7–6.9	**<0.001**	5.1	3.9–6.4	5.2	4.3–6.5	5.2	4.1–6.6	0.659
Copper (mg)	0.4	0.3–0.5	0.5	0.4–0.7	0.6	0.5–0.8	**<0.001**	0.5	0.4–0.6	0.5	0.4–0.7	0.5	0.4–0.8	**0.032**
Manganese (mg)	1.4	1.0–2.0	1.8	1.2–2.4	1.9	1.4–2.4	**<0.001**	1.5	1.1–2.1	1.7	1.2–2.4	1.8	1.2–2.3	0.058
Iodine (µg)	80.7	56.3–111.0	89.1	64.1–115.5	98.8	66.9–116.3	0.129	87.4	62.3–113.2	87.3	61.5–113.3	87.5	66.4–114.6	0.972
**Vitamins**														
Vitamin A (µg)	751.0	540.4–1055.3	806.8	561.1–1097.7	787.0	629.3–1099.4	0.318	815.6	577.3–1183.6	757.3	541.5–1066.3	744.7	571.3–995.7	0.388
Retinol (µg)	143.0	55.9–225.5	218.0	155.3–281.6	244.6	165.8–311.0	**<0.001**	160.1	77.6–252.6	216.2	146.3–285.1	204.7	148.0–300.7	**<0.001**
β-carotene (µg)	2600.4	1578.0–4447.4	2918.5	1751.0–4477.7	2840.9	1650.4–4930.1	0.707	3049.6	1807.7–4945.6	2612.2	1441.3–4353.2	2636.5	1723.8–4082.2	0.175
Vitamin D (µg)	3.9	1.1–8.8	2.2	1.1–6.7	2.2	1.3–4.9	0.348	3.3	1.2–8.7	2.2	1.2–5.9	2.0	1.0–3.5	**0.045**
Vitamin E (mg)	4.7	3.4–6.3	4.8	3.6–6.4	5.6	3.9–6.9	0.083	4.9	3.5–6.7	4.9	3.8–6.1	5.0	3.5–7.3	0.759
Vitamin B_1 (mg)	0.5	0.4–0.7	0.6	0.5–0.8	0.7	0.6–0.8	**<0.001**	0.5	0.4–0.7	0.6	0.5–0.8	0.6	0.5–0.7	**0.010**
Vitamin B_2 (mg)	1.1	0.8–1.4	1.1	0.9–1.5	1.3	1.1–1.6	**<0.001**	1.1	0.8–1.4	1.2	0.9–1.5	1.1	0.9–1.5	0.167
Niacin (mg)	6.7	4.4–9.2	7.9	6.3–10.4	9.1	6.8–11.3	**<0.001**	7.3	5.3–9.5	7.8	5.9–10.2	7.8	6.2–10.9	0.190
Vitamin B_6 (mg)	0.9	0.7–1.1	1.1	0.8–1.3	1.2	1.0–1.4	**<0.001**	0.9	0.8–1.2	1.0	0.8–1.3	1.1	0.8–1.4	0.136
Folates (µg)	143.6	114.2–176.4	161.8	127.9–195.9	178.5	149.3–200.7	**<0.001**	154.9	120.1–190.0	161.0	127.7–197.7	160.9	123.1–184.5	0.593
Vitamin B_{12} (µg)	1.9	1.3–2.5	2.2	1.6–2.9	2.5	2.0–3.1	**<0.001**	2.1	1.4–2.7	2.2	1.7–2.8	2.1	1.5–2.9	0.149
Vitamin C (mg)	61.2	41.6–86.0	64.1	43.8–89.0	65.4	46.2–91.4	0.707	65.1	47.8–87.0	63.7	44.9–88.2	48.5	34.6–91.4	0.214

Child-pHDS—Children's pro-Healthy Diet Score developed for children aged 13-72 months (low: 0-2 points, moderate: 3-5 points, high: 6-7 points), Child-nHDS—Children's non-Healthy Diet Score developed for children aged 13-72 months (low: 0-3 points, moderate: 4-7 points, high: 8-10 points), p—significance level of Kruskal-Wallis test with FDR correction, E—daily energy intake, SFA—saturated fatty acids, MUFA—monounsaturated fatty acids, PUFA—polyunsaturated fatty acids, LA—linoleic acid, ALA—α-linolenic acid, EPA—eicosapentaenoic acid, DHA—docosahexaenoic acid.

Table S6. Median values (Me) with lower and upper quartile ranges (Q1-Q3) of daily energy and nutrient intake by the diet quality levels in children aged 37-72 months (n=300)

Variables	Child-pHDS							Child-nHDS						
	Low (n=115)		Moderate (n=142)		High (n=43)		p	Low (n=126)		Moderate (n=133)		High (n=41)		p
	Me	Q1-Q3	Me	Q1-Q3	Me	Q1-Q3		Me	Q1-Q3	Me	Q1-Q3	Me	Q1-Q3	
**Energy (kcal)**	1209.9	1049.4–1406.3	1283.0	1075.1–1457.7	1325.6	1076.3–1561.2	0.365	1240.3	1067.9–1406.3	1279.5	1099.2–1517.9	1176.0	1009.1–1467.0	0.609
**Protein**														
Protein (g)	48.2	40.9–57.6	50.6	42.5–61.0	52.9	42.2–65.6	0.332	49.3	43.0–58.8	51.0	42.0–59.4	49.8	39.9–61.0	0.889
Protein (%E)	15.6	13.8–17.4	15.6	14.3–17.1	16.1	14.8–17.6	0.571	16.2	14.4–17.7	15.4	14.0–16.9	16.1	15.2–16.8	0.520
Animal protein (g)	31.9	25.2–40.2	33.6	27.4–43.0	37.2	28.3–46.1	0.365	32.4	27.4–41.8	34.6	27.8–40.5	32.4	25.3–43.3	0.876
Plant protein (g)	15.3	13.0–17.6	16.2	12.6–19.1	16.4	13.3–19.3	0.365	15.7	13.1–18.4	15.7	13.0–18.8	15.7	12.3–17.7	0.972
**Fats**														
Fat (g)	39.6	31.7–48.7	42.2	35.1–50.8	42.9	34.0–53.1	0.365	41.9	33.1–50.1	42.2	35.9–50.8	39.6	32.2–49.7	0.757
Fat (%E)	29.2	26.2–33.5	29.8	26.3–33.8	30.0	26.7–33.9	0.884	30.0	26.6–33.8	29.6	26.6–33.6	28.4	24.8–33.8	0.834
SFA (g)	16.6	12.7–21.3	17.1	14.5–21.1	18.1	13.9–22.1	0.567	16.8	13.3–21.0	17.4	14.9–21.5	16.0	13.7–20.6	0.757
SFA (%E)	12.1	10.7–13.9	12.2	10.9–14.2	12.4	10.2–14.2	0.935	12.4	10.8–14.0	12.2	10.7–14.3	11.8	10.8–13.4	0.876
MUFA (g)	15.5	12.3–18.8	15.6	13.2–20.0	16.6	12.7–20.2	0.436	15.7	12.5–19.9	15.7	13.7–20.2	15.0	11.8–19.4	0.714
PUFA (g)	4.7	3.7–6.3	5.1	4.1–6.7	5.0	4.1–6.6	0.485	4.9	4.1–6.3	5.0	4.0–6.6	4.5	3.6–6.1	0.768
LA (g)	3.7	2.9–5.0	3.8	3.1–5.1	3.9	3.1–5.2	0.595	3.7	3.0–4.9	3.9	3.1–5.1	3.8	2.9–4.8	0.876
LA (%E)	2.8	2.3–3.3	2.8	2.4–3.4	2.9	2.3–3.4	0.935	2.9	2.4–3.4	2.7	2.3–3.3	2.8	2.5–3.2	0.876
ALA (g)	0.8	0.6–1.1	0.8	0.6–1.2	0.9	0.7–1.1	0.645	0.9	0.6–1.1	0.8	0.6–1.2	0.7	0.5–1.0	0.520
ALA (%E)	0.6	0.5–0.8	0.6	0.4–0.8	0.6	0.5–0.8	0.935	0.6	0.5–0.8	0.6	0.4–0.8	0.6	0.4–0.7	0.520
EPA (mg)	5.5	2.7–13.9	5.4	1.9–15.8	5.5	1.5–14.1	0.990	6.4	2.5–18.2	5.2	2.5–12.2	4.7	1.3–8.4	0.520
DHA (mg)	29.5	16.2–55.0	37.6	18.1–63.5	38.7	21.9–62.3	0.365	38.4	19.3–68.0	36.6	18.7–58.2	28.5	15.2–41.0	0.520
EPA + DHA (mg)	36.7	21.8–63.6	45.2	20.5–80.7	46.2	29.2–63.4	0.505	45.6	23.3–81.0	41.1	21.9–68.2	30.0	19.0–49.4	0.520
**Carbohydrates**														
Carbohydrates (g)	164.9	146.4–203.0	174.0	149.6–211.3	178.8	151.4–206.1	0.511	168.9	145.0–203.3	176.9	153.7–211.0	163.3	143.5–216.7	0.520
Carbohydrates (%E)	52.9	49.3–56.6	52.7	47.9–56.3	51.7	49.1–54.8	0.645	52.0	47.7–55.5	53.1	49.3–56.7	52.9	49.1–58.1	0.664
Starch (g)	85.0	68.0–101.2	85.4	69.0–100.9	91.9	77.0–103.9	0.365	82.7	68.4–100.1	86.2	71.3–102.5	86.2	73.8–101.6	0.772
Lactose (g)	11.0	7.1–16.1	12.0	8.1–17.2	13.2	9.0–17.9	0.365	11.6	7.7–16.7	12.5	8.3–16.9	11.3	7.8–16.5	0.834
Sucrose (g)	36.3	27.4–47.5	36.0	25.1–47.0	33.6	24.5–39.7	0.365	31.7	20.9–42.7	37.7	29.3–48.2	32.9	25.1–43.5	0.282
Sucrose (%E)	12.1	9.2–14.4	11.0	8.7–13.6	10.1	8.3–11.3	0.107	10.1	7.5–13.6	11.5	9.6–14.0	10.9	8.7–13.0	0.439
Fibre (g)	10.2	8.0–12.9	11.1	9.3–14.0	11.2	10.1–14.8	0.110	10.9	8.9–14.3	10.8	9.0–14.0	9.8	8.0–12.2	0.520

Variables	Child-pHDS							Child-nHDS						
	Low (n=115)		Moderate (n=142)		High (n=43)		p	Low (n=126)		Moderate (n=133)		High (n=41)		p
	Me	Q1-Q3	Me	Q1-Q3	Me	Q1-Q3		Me	Q1-Q3	Me	Q1-Q3	Me	Q1-Q3	
**Minerals**														
Sodium (mg)	2003.9	1662.2–2561.3	2057.3	1715.8–2380.1	2239.1	1597.9–2599.3	0.595	2011.3	1710.8–2382.3	2069.9	1719.5–2569.6	2139.8	1708.7–2399.3	0.785
Potassium (mg)	1934.2	1585.2–2292.7	2057.1	1682.3–2530.0	2026.0	1768.6–2425.6	0.227	1989.0	1651.9–2401.2	2014.0	1687.4–2457.3	1893.5	1571.2–2176.4	0.570
Calcium (mg)	530.7	407.7–661.1	572.4	471.1–738.5	550.7	471.3–809.2	0.146	561.1	439.7–735.1	557.6	455.5–687.2	539.1	440.7–603.2	0.834
Phosphorus (mg)	773.0	636.1–921.5	828.6	703.4–1 006.7	885.0	742.4–1 027.3	0.107	815.5	682.6–971.5	809.1	670.0–966.5	800.4	621.7–918.4	0.785
Magnesium (mg)	177.0	142.5–212.6	187.6	149.6–222.5	191.3	164.1–242.6	0.222	185.0	150.8–223.4	185.5	156.8–224.3	182.8	135.8–211.9	0.714
Iron (mg)	5.7	4.8–7.3	6.5	5.1–7.7	6.8	5.5–8.4	0.107	6.2	5.0–7.6	6.5	5.1–7.7	5.6	4.8–7.1	0.520
Zinc (mg)	5.3	4.5–6.8	5.8	4.8–6.9	6.0	5.3–7.8	0.107	5.7	4.8–7.0	5.8	4.6–6.9	5.3	4.3–6.5	0.609
Copper (mg)	0.6	0.5–0.8	0.7	0.5–0.8	0.7	0.6–0.8	0.110	0.7	0.5–0.8	0.7	0.5–0.8	0.6	0.5–0.8	0.609
Manganese (mg)	2.2	1.8–2.9	2.3	1.7–2.9	2.5	2.0–3.0	0.578	2.4	1.8–2.9	2.3	1.8–2.9	2.2	1.8–2.8	0.876
Iodine (μg)	82.4	65.2–103.9	85.8	68.0–99.1	90.6	78.2–115.2	0.365	86.4	66.6–107.3	85.6	67.9–102.9	82.3	65.2–96.3	0.834
**Vitamins**														
Vitamin A (μg)	714.3	492.8–950.5	801.2	485.0–1024.7	762.5	574.6–957.5	0.365	759.1	457.8–1187.3	765.7	568.9–957.5	736.8	492.8–900.3	0.785
Retinol (μg)	264.9	181.2–321.1	259.1	203.6–335.5	283.3	207.2–456.0	0.365	266.1	192.9–334.2	268.7	210.4–344.5	222.8	193.2–303.6	0.570
β-carotene (μg)	2492.8	1458.4–3723.9	3144.2	1401.5–4654.6	2597.9	1740.1–3550.7	0.518	2663.8	1445.7–4788.5	2597.9	1701.1–3708.5	2541.0	1308.4–3762.3	0.876
Vitamin D (μg)	1.3	0.8–1.8	1.3	1.0–2.0	1.6	1.1–2.1	0.332	1.3	0.9–2.2	1.4	1.0–1.9	1.2	0.8–1.8	0.609
Vitamin E (mg)	4.5	3.6–5.9	5.2	4.0–6.8	5.1	4.0–6.5	0.269	5.0	4.0–6.8	4.9	3.9–6.4	4.4	3.3–5.8	0.520
Vitamin B_1 (mg)	0.7	0.6–0.9	0.7	0.6–0.9	0.8	0.6–0.9	0.269	0.7	0.6–0.8	0.7	0.6–0.9	0.7	0.5–0.9	0.643
Vitamin B_2 (mg)	1.1	0.9–1.4	1.2	1.0–1.5	1.3	1.0–1.6	0.107	1.2	1.0–1.5	1.2	1.0–1.5	1.2	0.9–1.4	0.714
Niacin (mg)	10.1	6.9–12.7	9.3	7.6–12.6	9.9	7.4–13.7	0.935	10.0	7.7–11.9	9.4	7.4–13.4	9.8	6.8–12.9	0.876
Vitamin B_6 (mg)	1.1	0.9–1.3	1.2	1.0–1.5	1.2	1.0–1.4	0.332	1.2	1.0–1.4	1.2	1.0–1.5	1.1	0.9–1.3	0.609
Folates (μg)	152.8	126.9–186.5	172.8	141.7–217.4	163.9	133.5–210.1	0.107	162.1	136.8–213.1	165.0	134.3–208.9	156.5	116.5–183.3	0.520
Vitamin B_{12} (μg)	2.1	1.6–2.6	2.3	1.8–2.8	2.3	2.0–3.1	0.107	2.3	1.7–3.0	2.2	1.8–2.7	2.0	1.8–2.8	0.834
Vitamin C (mg)	49.8	33.9–68.3	55.2	40.3–86.7	53.1	36.6–74.9	0.269	57.1	37.5–83.2	53.1	38.7–75.2	45.4	25.5–62.6	0.439

Child-pHDS—Children's pro-Healthy Diet Score developed for children aged 13-72 months (low: 0-2 points, moderate: 3-5 points, high: 6-7 points), Child-nHDS—Children's non-Healthy Diet Score developed for children aged 13-72 months (low: 0-3 points, moderate: 4-7 points, high: 8-10 points), p—significance level of Kruskal-Wallis test with FDR correction, E—daily energy intake, SFA—saturated fatty acids, MUFA—monounsaturated fatty acids, PUFA—polyunsaturated fatty acids, LA—linoleic acid, ALA—α-linolenic acid, EPA—eicosapentaenoic acid, DHA—docosahexaenoic acid.

Table S7. Percentage of children (%) at risk of inadequate nutrient intake according to the dietary recommendations by the diet quality levels in children aged 13-36 months (n=400)

Variables	Criterion	Child-pHDS				Child-nHDS			
		Low (n=155)	Moderate (n=180)	High (n=65)	p	Low (n=174)	Moderate (n=169)	High (n=57)	p
		%	%	%		%	%	%	
**Protein**									
Protein (g)	<AI or EAR[1]	6.5	0.6	0.0	**0.008**	4.0	2.4	0.0	0.437
Protein (%E)	< AMDR	1.3	0.6	0.0	0.621	0.6	1.2	0.0	0.679
Protein (%E)	> AMDR	29.7	28.9	29.2	0.979	35.1	25.4	22.8	0.231
**Fats**									
Fat (%E)	< AMDR	88.4	83.9	80.0	0.231	86.2	84.0	84.2	0.346
Fat (%E)	> AMDR	5.8	4.4	7.7	0.414	7.5	3.0	7.0	0.111
SFA (%E)	≥10%	53.5	73.9	72.3	**0.005**	58.0	72.8	68.4	0.088
LA (%E)	<AI	89.0	89.4	87.7	0.957	89.1	91.1	82.5	0.421
ALA (%E)	<AI	39.4	37.2	40.0	0.947	42.5	37.9	28.1	0.346
EPA + DHA (mg)	<AI	92.9	84.4	81.5	0.056	84.5	89.3	89.5	0.495
**Carbohydrates**									
Carbohydrates (%E)	< AMDR	8.4	9.4	16.9	0.303	11.5	7.7	14.0	0.437
Carbohydrates (%E)	> AMDR	9.0	1.1	1.5	**0.005**	7.5	1.8	1.8	0.088
Sucrose (%E)	≥10%	51.0	48.3	46.2	0.865	41.4	53.8	57.9	0.111
Fibre (g)	<AI	65.2	44.4	36.9	**0.005**	54.0	49.1	49.1	0.679
**Minerals**									
Sodium (mg)	>AI	77.4	92.8	95.4	**0.005**	80.5	92.3	93.0	**0.033**
Potassium (mg)	<AI	3.9	1.1	3.1	0.387	3.4	1.8	1.8	0.668
Calcium (mg)	<AI or EAR[1]	49.7	43.9	30.8	0.085	48.9	39.6	42.1	0.423
Phosphorus (mg)	<AI or EAR[1]	14.2	5.0	4.6	**0.016**	11.5	7.1	3.5	0.327
Magnesium (mg)	<AI or EAR[1]	6.5	1.7	3.1	0.140	5.2	3.0	1.8	0.495
Iron (mg)	<AI or EAR[2]	7.1	2.2	4.6	0.194	7.5	1.8	3.5	0.125
Zinc (mg)	<AI or EAR[2]	7.7	2.2	3.1	0.097	8.6	1.2	1.8	**0.033**
Copper (mg)	<AI or EAR[1]	12.3	3.9	3.1	**0.016**	9.2	5.9	3.5	0.437
Manganese (mg)	<AI	38.1	25.0	20.0	**0.018**	33.3	26.0	26.3	0.454
Iodine (μg)	<AI or EAR[1]	34.8	27.2	24.6	0.303	29.9	31.4	24.6	0.679

Variables	Criterion	Child-pHDS				Child-nHDS			
		Low (n=155)	Moderate (n=180)	High (n=65)	p	Low (n=174)	Moderate (n=169)	High (n=57)	p
		%	%	%		%	%	%	
**Vitamins**									
Vitamin A (μg)	<AI or EAR[1]	2.6	1.1	0.0	0.414	0.6	2.4	1.8	0.495
Vitamin D (μg)	<AI	94.8	95.0	98.5	0.552	94.3	97.0	94.7	0.538
Vitamin E (mg)	<AI	70.3	67.8	60.0	0.432	64.9	72.2	61.4	0.421
Vitamin B_1 (mg)	<AI or EAR[1]	24.5	8.9	6.2	**0.005**	20.7	11.2	5.3	0.055
Vitamin B_2 (mg)	<AI or EAR[1]	5.2	2.8	0.0	0.231	5.7	1.8	0.0	0.125
Niacin (mg)	<AI or EAR[1]	30.3	13.3	9.2	**0.005**	22.4	16.0	19.3	0.479
Vitamin B_6 (mg)	<AI or EAR[1]	3.2	1.1	1.5	0.471	2.9	1.8	0.0	0.495
Folates (μg)	<AI or EAR[1]	31.6	18.3	12.3	**0.005**	23.6	21.9	21.1	0.897
Vitamin B_{12} (μg)	<AI or EAR[1]	7.7	2.2	1.5	0.056	7.5	2.4	0.0	0.088
Vitamin C (mg)	<AI or EAR[1]	12.9	12.2	7.7	0.621	12.6	10.1	14.0	0.679

Child-pHDS—Children's pro-Healthy Diet Score developed for children aged 13-72 months (low: 0-2 points, moderate: 3-5 points, high: 6-7 points), Child-nHDS—Children's non-Healthy Diet Score developed for children aged 13-72 months (low: 0-3 points, moderate: 4-7 points, high: 8-10 points), p—significance level of chi^2 test with FDR correction, E—daily energy intake, AI—adequate intake, EAR—estimated average requirement, AMDR—acceptable macronutrient distribution range, SFA—saturated fatty acids, LA—linoleic acid, ALA—α-linolenic acid, EPA—eicosapentaenoic acid, DHA—docosahexaenoic acid, [1] depending on the child age: <AI for children aged 5-12 months and <EAR for children aged 13-72 months, [2] <AI for children aged 5-6 months and <EAR for children aged 7-72 months.

Table S8. Percentage of children (%) at risk of inadequate nutrient intake according to the dietary recommendations by the diet quality levels in children aged 37-72 months (n=300)

Variables	Criterion	Child-pHDS				Child-nHDS			
		Low (n=115)	Moderate (n=142)	High (n=43)	p	Low (n=126)	Moderate (n=133)	High (n=41)	p
		%	%	%		%	%	%	
**Protein**									
Protein (g)	<AI or EAR[1]	0.0	0.0	0.0	1.000	0.0	0.0	0.0	1.000
Protein (%E)	< AMDR	0.0	0.0	0.0	1.000	0.0	0.0	0.0	1.000
Protein (%E)	> AMDR	7.0	6.3	4.7	0.989	10.3	3.0	4.9	0.264
**Fats**									
Fat (%E)	< AMDR	2.6	1.4	2.3	0.975	2.4	2.3	0.0	0.765
Fat (%E)	> AMDR	13.9	19.0	14.0	0.954	19.0	12.8	19.5	0.622
SFA (%E)	≥10%	85.2	85.9	76.7	0.954	86.5	83.5	80.5	0.765
LA (%E)	<AI	95.7	93.7	93.0	0.975	92.9	94.7	97.6	0.752
ALA (%E)	<AI	29.6	31.7	25.6	0.975	23.0	33.8	39.0	0.272
EPA + DHA (mg)	<AI	94.8	93.0	95.3	0.975	90.5	95.5	100.0	0.264
**Carbohydrates**									
Carbohydrates (%E)	< AMDR	8.7	9.9	14.0	0.954	11.1	6.8	17.1	0.410
Carbohydrates (%E)	> AMDR	1.7	1.4	2.3	0.991	1.6	2.3	0.0	0.765
Sucrose (%E)	≥10%	70.4	57.0	51.2	0.352	50.0	71.4	63.4	0.066
Fibre (g)	<AI	79.1	74.6	69.8	0.954	73.0	74.4	87.8	0.410
**Minerals**									
Sodium (mg)	>AI	98.3	99.3	100.0	0.954	98.4	99.2	100.0	0.765
Potassium (mg)	<AI	1.7	2.8	0.0	0.954	2.4	0.8	4.9	0.563
Calcium (mg)	<AI or EAR[1]	93.0	82.4	74.4	0.165	81.7	88.7	85.4	0.584
Phosphorus (mg)	<AI or EAR[1]	1.7	0.7	0.0	0.954	1.6	0.8	0.0	0.765
Magnesium (mg)	<AI or EAR[1]	4.3	4.9	0.0	0.954	4.0	1.5	12.2	0.148
Iron (mg)	<AI or EAR[2]	7.8	7.7	2.3	0.954	6.3	5.3	14.6	0.373
Zinc (mg)	<AI or EAR[2]	13.0	9.9	4.7	0.954	8.7	9.8	17.1	0.584
Copper (mg)	<AI or EAR[1]	0.9	0.0	0.0	0.954	0.0	0.0	2.4	0.264
Manganese (mg)	<AI	9.6	19.0	11.6	0.490	14.3	12.0	22.0	0.584
Iodine (μg)	<AI or EAR[1]	24.3	21.1	18.6	0.975	22.2	21.1	24.4	0.990

Variables	Criterion	Child-pHDS				Child-nHDS			
		Low (n=115)	Moderate (n=142)	High (n=43)	p	Low (n=126)	Moderate (n=133)	High (n=41)	p
		%	%	%		%	%	%	
**Vitamins**									
Vitamin A (μg)	<AI or EAR[1]	3.5	2.1	2.3	0.975	4.0	2.3	0.0	0.629
Vitamin D (μg)	<AI	100.0	100.0	100.0	1.000	100.0	100.0	100.0	1.000
Vitamin E (mg)	<AI	75.7	65.5	65.1	0.816	67.5	68.4	78.0	0.696
Vitamin B_1 (mg)	<AI or EAR[1]	15.7	14.8	9.3	0.954	14.3	11.3	24.4	0.373
Vitamin B_2 (mg)	<AI or EAR[1]	0.9	0.0	0.0	0.954	0.8	0.0	0.0	0.752
Niacin (mg)	<AI or EAR[1]	13.0	10.6	11.6	0.975	10.3	12.8	12.2	0.934
Vitamin B_6 (mg)	<AI or EAR[1]	0.0	0.7	0.0	0.954	0.0	0.0	2.4	0.264
Folates (μg)	<AI or EAR[1]	53.9	40.1	46.5	0.490	46.8	43.6	53.7	0.752
Vitamin B_{12} (μg)	<AI or EAR[1]	5.2	0.7	0.0	0.352	4.0	1.5	0.0	0.563
Vitamin C (mg)	<AI or EAR[1]	38.3	24.6	27.9	0.479	27.8	27.8	46.3	0.264

Child-pHDS—Children's pro-Healthy Diet Score developed for children aged 13-72 months (low: 0-2 points, moderate: 3-5 points, high: 6-7 points), Child-nHDS—Children's non-Healthy Diet Score developed for children aged 13-72 months (low: 0-3 points, moderate: 4-7 points, high: 8-10 points), p—significance level of chi^2 test with FDR correction, E—daily energy intake, AI—adequate intake, EAR—estimated average requirement, AMDR—acceptable macronutrient distribution range, SFA—saturated fatty acids, LA—linoleic acid, ALA—α-linolenic acid, EPA—eicosapentaenoic acid, DHA—docosahexaenoic acid, [1] depending on the child age: <AI for children aged 5-12 months and <EAR for children aged 13-72 months, [2] <AI for children aged 5-6 months and <EAR for children aged 7-72 months.

Table S9. Crude odds ratios (ORs, 95% CI) for the risk of inadequate nutrient intake according to the dietary recommendations by the diet quality levels in children aged 13-72 months

Variables	Criterion	Children aged 13-36 months				Children aged 37-72 months			
		Child-pHDS high *vs* low (ref.)		Child-nHDS high *vs* low (ref.)		Child-pHDS high *vs* low (ref.)		Child-nHDS high *vs* low (ref.)	
		OR_{crude}	95% CI	OR_{crude}	95% CI	OR_{crude}	95% CI	OR_{crude}	95% CI
**Protein**									
Protein (g)	<AI or EAR[1]	NA	NA	NA	NA	NA	NA	NA	NA
Protein (%E)	< AMDR	NA	NA	NA	NA	NA	NA	NA	NA
Protein (%E)	> AMDR	0.96	0.51–1.82	0.54	0.27–1.08	0.65	0.13–3.20	0.45	0.10–2.06
**Fats**									
Fat (%E)	< AMDR	0.43	0.16–1.17	0.70	0.23–2.13	0.89	0.09–8.82	NA	NA
Fat (%E)	> AMDR	0.63	0.15–2.66	0.68	0.15–3.16	1.00	0.36–2.76	1.00	0.41–2.44
SFA (%E)	≥10%	**2.27***	**1.21–4.25**	1.57	0.83–2.95	0.57	0.24–1.37	0.64	0.25–1.62
LA (%E)	<AI	0.88	0.36–2.15	0.58	0.25–1.32	0.61	0.14–2.65	3.08	0.38–25.05
ALA (%E)	<AI	1.03	0.57–1.86	0.53	0.27–1.01	0.82	0.37–1.81	**2.14***	**1.01–4.54**
EPA + DHA (mg)	<AI	**0.34***	**0.14–0.81**	1.56	0.61–4.00	1.13	0.22–5.82	NA	NA
**Carbohydrates**									
Carbohydrates (%E)	< AMDR	2.04	0.86–4.85	1.17	0.49–2.84	1.72	0.58–5.06	1.62	0.60–4.33
Carbohydrates (%E)	> AMDR	0.17	0.02–1.35	0.23	0.03–1.77	1.43	0.13–16.25	NA	NA
Sucrose (%E)	≥10%	0.82	0.46–1.47	**1.95***	**1.06–3.57**	**0.44***	**0.21–0.90**	1.73	0.84–3.58
Fibre (g)	<AI	**0.31*****	**0.17–0.57**	0.82	0.45–1.50	0.61	0.28–1.34	2.66	0.96–7.34
**Minerals**									
Sodium (mg)	>AI	**6.03****	**1.78–20.38**	**3.22***	**1.09–9.51**	NA	NA	NA	NA
Potassium (mg)	<AI	0.79	0.15–4.01	0.50	0.06–4.24	NA	NA	2.10	0.34–13.04
Calcium (mg)	<AI or EAR[1]	**0.45***	**0.24–0.83**	0.76	0.42–1.39	**0.22****	**0.08–0.59**	1.30	0.49–3.46
Phosphorus (mg)	<AI or EAR[1]	0.29	0.08–1.01	0.28	0.06–1.24	NA	NA	NA	NA
Magnesium (mg)	<AI or EAR[1]	0.46	0.10–2.16	0.33	0.04–2.64	NA	NA	3.36	0.92–12.26
Iron (mg)	<AI or EAR[2]	0.63	0.17–2.35	0.45	0.10–2.06	0.28	0.03–2.28	2.53	0.82–7.78
Zinc (mg)	<AI or EAR[2]	0.38	0.08–1.74	0.19	0.02–1.47	0.33	0.07–1.49	2.15	0.77–5.98
Copper (mg)	<AI or EAR[1]	0.23	0.05–1.01	0.36	0.08–1.61	NA	NA	NA	NA
Manganese (mg)	<AI	**0.41***	**0.20–0.81**	0.71	0.37–1.39	1.24	0.41–3.81	1.69	0.69–4.12
Iodine (μg)	<AI or EAR[1]	0.61	0.32–1.17	0.76	0.39–1.52	0.71	0.30–1.71	1.13	0.49–2.58

Variables	Criterion	Children aged 13-36 months				Children aged 37-72 months			
		Child-pHDS high *vs* low (ref.)		Child-nHDS high *vs* low (ref.)		Child-pHDS high *vs* low (ref.)		Child-nHDS high *vs* low (ref.)	
		OR_{crude}	95% CI	OR_{crude}	95% CI	OR_{crude}	95% CI	OR_{crude}	95% CI
**Vitamins**									
Vitamin A (µg)	<AI or EAR[1]	NA	NA	3.09	0.19–50.20	0.66	0.07–6.08	NA	NA
Vitamin D (µg)	<AI	3.48	0.43–28.43	1.10	0.29–4.13	NA	NA	NA	NA
Vitamin E (mg)	<AI	0.63	0.35–1.16	0.86	0.46–1.59	0.60	0.28–1.28	1.72	0.75–3.93
Vitamin B_1 (mg)	<AI or EAR[1]	**0.20****	**0.07–0.59**	**0.21***	**0.06–0.72**	0.55	0.18–1.74	1.94	0.81–4.62
Vitamin B_2 (mg)	<AI or EAR[1]	NA	NA	NA	NA	NA	NA	NA	NA
Niacin (mg)	<AI or EAR[1]	**0.23****	**0.09–0.58**	0.83	0.39–1.75	0.88	0.30–2.58	1.21	0.40–3.62
Vitamin B_6 (mg)	<AI or EAR[1]	0.47	0.05–4.09	NA	NA	NA	NA	NA	NA
Folates (µg)	<AI or EAR[1]	**0.30****	**0.13–0.69**	0.87	0.42–1.79	0.74	0.37–1.50	1.31	0.65–2.67
Vitamin B_{12} (µg)	<AI or EAR[1]	0.19	0.02–1.46	NA	NA	NA	NA	NA	NA
Vitamin C (mg)	<AI or EAR[1]	0.56	0.20–1.57	1.13	0.47–2.69	0.62	0.29–1.34	**2.25***	**1.09–4.65**

Child-pHDS—Children's pro-Healthy Diet Score developed for children aged 13-72 months (low: 0-2 points, high: 6-7 points), Child-nHDS—Children's non-Healthy Diet Score developed for children aged 13-72 months (low: 0-3 points, high: 8-10 points), OR—odds ratio, CI—confidence interval, E—daily energy intake, AI—adequate intake, EAR—estimated average requirement, AMDR—acceptable macronutrient distribution range, SFA—saturated fatty acids, LA—linoleic acid, ALA—α-linolenic acid, EPA—eicosapentaenoic acid, DHA—docosahexaenoic acid, [1] depending on the child age: <AI for children aged 5-12 months and <EAR for children aged 13-72 months, [2] <AI for children aged 5-6 months and <EAR for children aged 7-72 months; *p<0.05, **p<0.01, ***p<0.001 (significance level of Wald's test); NA—odds ratios are not available due to the zero values in contingency tables.
